# Large Language Model Translation of BI-RADS Breast Imaging Reports into Arabic: A Blinded Expert Evaluation of Diagnostic Communication Safety

**DOI:** 10.3390/diagnostics16162495

**Published:** 2026-08-07

**Authors:** Mohammad Alarifi, Jake Luo, Abdulrahman Jabour, Yazeed Alashban, Meaad Almusined, Maram Mobara, Alhanouf Alshedi, Mansour Almanaa

**Affiliations:** 1 Radiological Sciences Department, College of Applied Medical Sciences, King Saud University, Riyadh 4545, Saudi Arabia; mohalarifi@ksu.edu.sa (M.A.); yalashban@ksu.edu.sa (Y.A.); malmusined@ksu.edu.sa (M.A.); aalshedi@ksu.edu.sa (A.A.); 2 College of Health Sciences, University of Wisconsin–Milwaukee, Milwaukee, WI 53211, USA; jakeluo@uwm.edu; 3 Health Informatics Department, Faculty of Public Health and Tropical Medicine, Jazan University, Jazan 45142, Saudi Arabia; ajabour@jazanu.edu.sa; 4 Radiology Department, Prince Sultan Military Medical City, Riyadh 12233, Saudi Arabia; dr.mobara@gmail.com

**Keywords:** large language models, breast imaging reports, BI-RADS, Arabic translation, patient-centered radiology, diagnostic communication, clinical safety

## Abstract

**Background/Objectives:** Breast imaging reports contain Breast Imaging Reporting and Data System (BI-RADS) assessments and management recommendations that may be difficult for patients to understand across languages. Large language models (LLMs) may support patient-facing communication, but clinically important details must be preserved. This study compared radiologists’ opinions regarding the quality, clinical fidelity, safety of wording, and communication usefulness of patient-friendly Arabic translations of BI-RADS breast imaging reports generated by three LLMs. **Methods:** Five de-identified reports representing BI-RADS categories 0, 2, 3, 4, and 6 were translated from English into Arabic by DeepSeek, ChatGPT, and Gemini using an identical structured prompt. Fifty radiologists rated the blinded outputs across eight 5-point domains. Model ratings were compared using Friedman tests, Kendall’s W, and multiplicity-adjusted Wilcoxon signed-rank tests. Laterality was also verified against the source reports. **Results:** Gemini achieved the highest overall mean score (3.73 ± 0.78), followed by DeepSeek (3.54 ± 0.73) and ChatGPT (3.03 ± 0.70). The overall model effect was significant (χ^2^ = 34.11, df = 2, *p* < 0.001; Kendall’s W = 0.341). Gemini and DeepSeek each outperformed ChatGPT across all eight domains (adjusted *p* < 0.001), and Gemini outperformed DeepSeek overall (adjusted *p* = 0.009). No laterality errors were identified among the 15 translations. **Conclusions:** Performance remained model-dependent despite the shared prompt. Among the participating radiologists, Gemini received the highest expert ratings, while DeepSeek remained competitive. Because errors involving BI-RADS categories, laterality, measurements, lesion location, or recommendations could change diagnostic understanding, LLM-generated Arabic translations should serve as radiologist-reviewed communication aids rather than autonomous substitutes for clinical explanation.

## 1. Introduction

Radiology reports are written primarily for communication among clinicians and commonly contain specialized terminology, structured descriptors, and management recommendations that are not readily accessible to patients [1]. As electronic portals increasingly provide patients with direct access to imaging results, the gap between information access and information comprehension has become more visible [2]. Evidence from report simplification research suggests that LLM-generated patient-friendly versions can improve readability and patient-perceived understanding, but occasional factual errors and potentially harmful wording remain important barriers to autonomous deployment [3,4,5,6].

These challenges are especially relevant to breast imaging. Mammography, ultrasound, and breast MRI reports frequently include laterality, lesion size, location, density classification, Breast Imaging Reporting and Data System (BI-RADS) assessment, and follow-up or biopsy recommendations. BI-RADS categories directly communicate the level of diagnostic certainty and guide subsequent care; consequently, a translation that alters a category, recommendation, measurement, or side of the breast can generate false reassurance, unnecessary anxiety, or inappropriate delay in follow-up [7,8]. Breast imaging also carries a substantial emotional burden because women may seek to interpret findings before or after consultation with surgeons, oncologists, primary care clinicians, or breast imaging teams [9].

Arabic-speaking women who have migrated to or been resettled in Western countries may face linguistic and health system barriers that affect access to breast cancer screening, follow-up care, and understanding of breast-related medical information [10,11]. Research involving Arab immigrant and refugee women has identified limited English fluency, unfamiliarity with the receiving country’s health system, and limited access to culturally and linguistically concordant communication as important barriers to breast cancer prevention and timely care [10]. In a qualitative study of Arabic-speaking refugee women in the United States, participants described language barriers, difficulty navigating health services, and concerns about interpreter accuracy and dialectal differences, while emphasizing the value of clear explanations from health professionals. Although these studies focused primarily on cancer screening rather than interpretation of radiology reports, they underscore the importance of accessible and culturally appropriate Arabic communication when women receive breast imaging results [10,11].

Large language models (LLMs) could offer fast, scalable translation and simplification, but multilingual performance is variable across models, target languages, medical terminology, and prompt design. Recent radiology translation studies have reported strong performance for some language pairs but clinically relevant omissions, literal translations, abbreviation errors, and potential harms in others [12,13,14]. In a broader scoping review of radiology report applications, performance was also reported to vary by model and language, with Arabic identified as a language requiring further targeted assessment [15].

Prompt design may help constrain outputs. Prior work has shown that detailed prompts can improve the retention of clinically relevant content, although model outputs remain stochastic and can over-simplify or omit information [16]. In breast imaging, a clinically safe prompt must do more than request plain language: it should preserve structured report sections, measurements, laterality, BI-RADS labels, and the original recommendation. Our study therefore compared Arabic translations generated using a structured prompt designed to produce patient-friendly language. We hypothesized that the tools would differ in expert-rated translation quality, clinical safety, and usefulness for clinical communication.

## 2. Materials and Methods

### 2.1. Study Design and Ethics

This cross-sectional study used a blinded expert evaluation survey design to compare Arabic translations generated using a standardized prompt intended to produce patient-friendly text. Before use in this study, all reports were de-identified, with patient names, medical record numbers, dates, and other potentially identifying information removed. The original reports and the blinded LLM-generated translations were presented to participants through an online Qualtrics survey platform (Qualtrics, Provo, UT, USA) for evaluation.

The University of Wisconsin–Milwaukee Institutional Review Board determined that the protocol (IRB #20.230) was exempt under Category 2 of 45 CFR 46.104(d). The study focused on radiologist assessments of the linguistic quality, preservation of clinical information, patient-friendly presentation, safety of wording, and communication usefulness of Arabic translations derived from breast imaging reports. The LLMs were evaluated only as tools for translating and simplifying the original report content; the original radiology findings, BI-RADS assessments, and management recommendations remained the reference standard throughout the evaluation.

### 2.2. Case Selection and Clinical Rationale

Five representative breast imaging reports were selected to span BI-RADS categories 0, 2, 3, 4, and 6 (Supplementary Material S1). The cases encompassed clinically relevant differences in diagnostic uncertainty, management implications, and anticipated emotional impact. They included reports from mammography, breast ultrasound, and breast MRI, and contained commonly encountered findings and recommendations. BI-RADS 0 was selected to represent an incomplete assessment requiring additional evaluation; BI-RADS 2 represented benign findings; BI-RADS 3 represented probably benign findings requiring surveillance; BI-RADS 4 represented suspicious abnormalities for which biopsy may be considered; and BI-RADS 6 represented biopsy-proven malignancy. This spectrum was chosen because the clinical implications of mistranslation differ meaningfully across benign, indeterminate, suspicious, and confirmed malignant scenarios [7,8]. Detailed characteristics of the five reports, including modality, laterality, lesion type, measurement burden, word count, and recommendation, are summarized in Supplementary Material S2.

Case selection was additionally informed by routine breast imaging practice. Patients often seek clarification of report terminology before or after scheduled consultations, and explanations may be delivered by clinicians with different levels of breast imaging expertise. A patient-friendly Arabic version might therefore help bridge communication gaps, but it must not alter clinical content or independently introduce medical advice. The case set was intended to test translation performance across varying levels of report complexity while retaining the report elements most critical for safe communication: laterality, location, measurements, BI-RADS labels, and recommendations.

### 2.3. LLM Translation Procedure and Prompt Design

**Figure 1:**
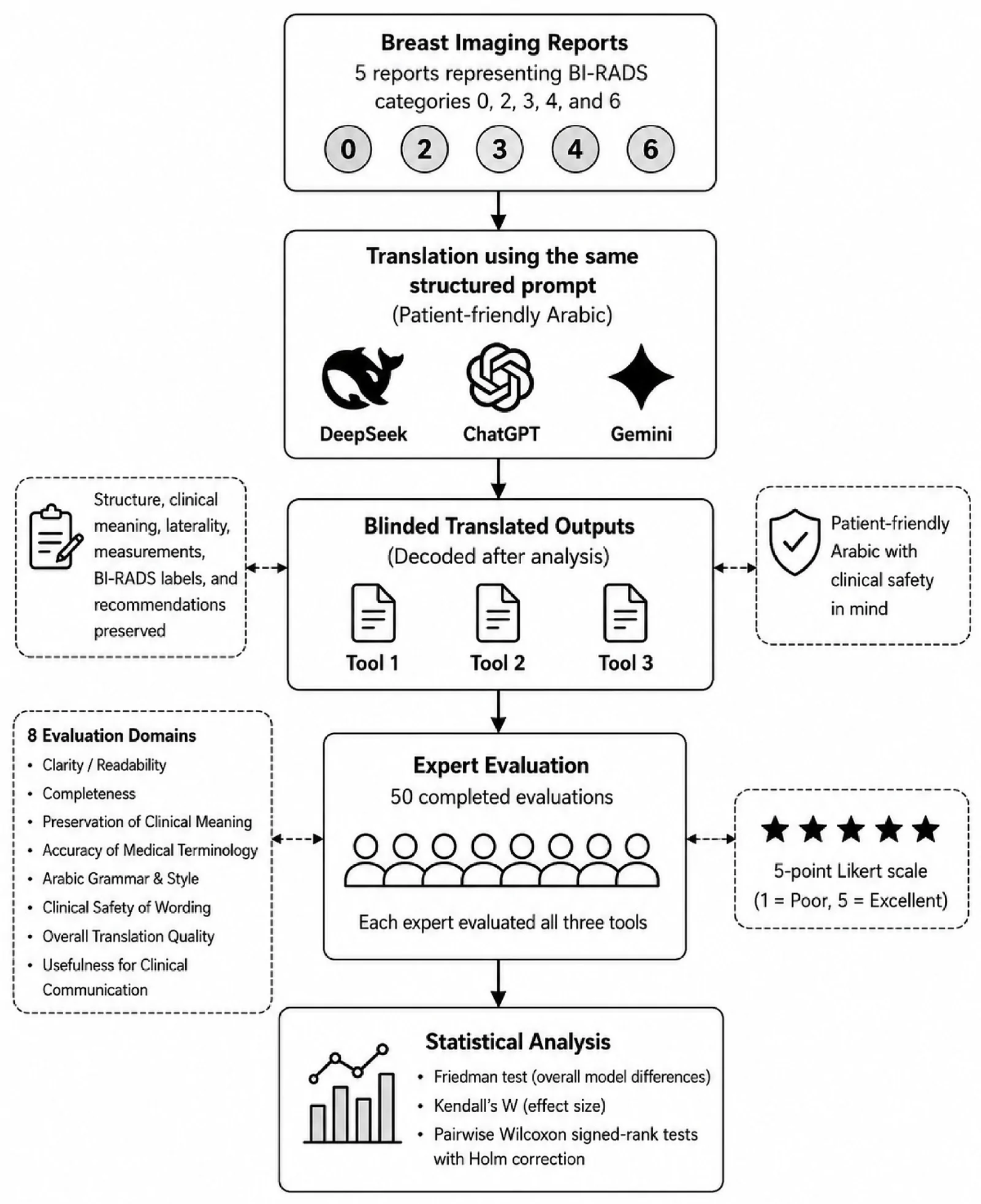
Study methodology. Five BI-RADS breast imaging reports were translated into Arabic using a prompt designed to produce patient-friendly language.

Each source report was translated from English into Arabic by three LLMs: DeepSeek (DeepSeek, Hangzhou, China), ChatGPT (OpenAI, San Francisco, CA, USA), and Gemini (Google, Mountain View, CA, USA) (Figure 1). All three received the same structured prompt (Supplementary Material S3). To ensure study reproducibility, translation tasks were conducted across three large language models via their primary web interfaces on 28 February 2026: ChatGPT (OpenAI; GPT-5), DeepSeek-V3 (DeepSeek), and Google Gemini (3.6 Flash/3.1 Pro). Each English breast imaging report was submitted into a brand-new chat session to prevent contextual memory bias or conversational drift across translation tasks. Input language settings were configured for English source text, with target output parameters set to standardized, patient-friendly Modern Standard Arabic. Because the models were accessed via standard web user interfaces, default system temperature/randomness parameters were maintained for each platform without manual adjustment. For each individual report prompt, all translations were selected directly from the first single generated response; no outputs were regenerated, multi-sampled, or selected from alternative attempts. Standardized system prompting instructed the models to strictly preserve clinical accuracy, report structural hierarchy, management recommendations, exact numerical measurements, laterality, and BI-RADS classification categories, while explicitly converting clock-face positions into clear anatomical location descriptions in Arabic without altering clinical meaning. The prompt assigned the model the roles of an experienced radiologist and an English–Arabic translator directed it to generate Arabic translations intended to improve readability for patients while preserving the original clinical meaning. The prompt required exact preservation of laterality, numeric values, measurements, dates, units, ACR breast density type, BI-RADS label, and recommendations. It further instructed the models to retain ambiguous abbreviations rather than infer an unsupported meaning. When a source report used clock-face locations, the model was directed to replace them with anatomically understandable Arabic location descriptions while maintaining the correct relation to the right or left breast. A standardized Arabic explanation could be placed after the original BI-RADS label, but only if it matched the category and did not introduce additional management advice. This design reflects evidence that structured prompts can improve clinical content retention while supporting clearer language [15,16]. Generation parameters such as random seed and temperature were not user-configurable through the publicly available web interfaces at the time of data collection; therefore, repeated generation experiments and within-model output variability were not evaluated.

### 2.4. Blinding, Evaluators, and Outcomes

Model outputs were relabeled as Tool 1, Tool 2, and Tool 3 before evaluation; the allocation was decoded only after statistical analysis (Tool 1 = DeepSeek, Tool 2 = ChatGPT, Tool 3 = Gemini). Fifty radiologists completed the evaluation. The sample included residents, registrars, fellows, and consultant radiologists; 49 of 50 evaluators (98.0%) reported Arabic as their native language, and 31 (62.0%) reported advanced English proficiency. Participant characteristics are presented in Table 1.

The questionnaire was developed based on quality dimensions discussed in prior studies [6,7,12,14,17], informing domains related to clarity, completeness, the preservation of clinical meaning, terminology accuracy, linguistic quality, clinical safety, and communication usefulness. Items were developed specifically for this study rather than adopted from a single existing validated instrument. The initial questionnaire was piloted with 10 radiologists (Pilot Study 1); based on their feedback and further discussion with breast imaging specialists, it was revised and re-evaluated in a second pilot (Pilot Study 2), with feedback incorporated into the final eight-item instrument used in the main study.

Each evaluator reviewed the blinded outputs and rated each tool across the eight domains. Ratings used a 5-point Likert scale, where 1 indicated poor performance and 5 indicated excellent performance. The primary outcome was the overall model score calculated from the ratings across the eight domains. The use of specialist evaluation was appropriate because patient-facing translations must be judged not only for fluency but also for fidelity to report content and safety-critical information [12,14,17].

### 2.5. Statistical Analysis

A retrospective power analysis was conducted using G*Power (version 3.1.9.7; Heinrich-Heine-Universität Düsseldorf, Düsseldorf, Germany) to contextualize the achieved sample size. Assuming a medium effect size (f = 0.25) per Cohen’s conventions, the repeated-measures ANOVA design (α = 0.05, power = 0.80, three within-subject measurements, assumed correlation among repeated measures = 0.5) required a minimum of 28 evaluators. The study’s final sample of 50 evaluators exceeded this threshold.

**Figure 2:**
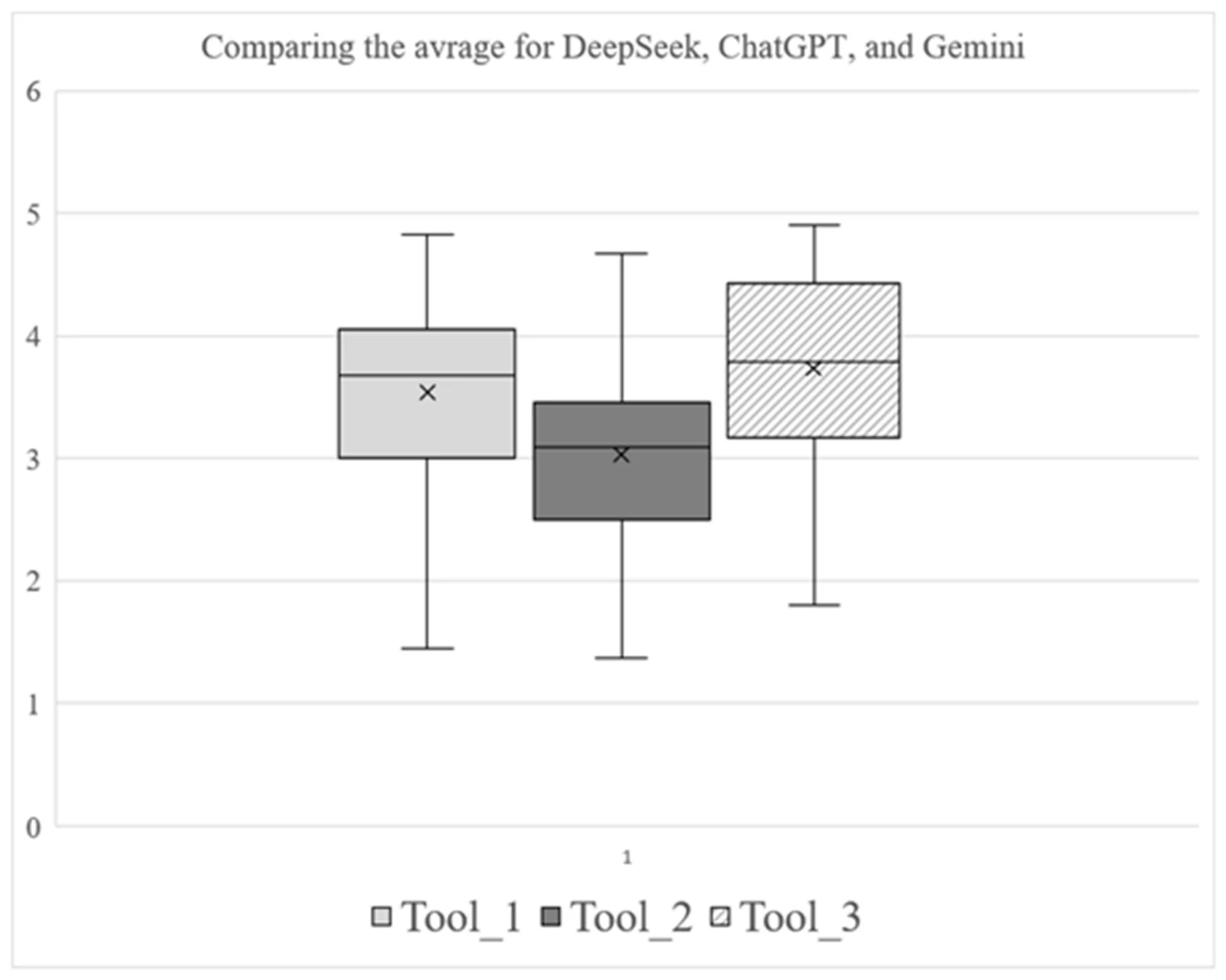
A boxplot of overall average translation quality scores for the three tools. Tool 1 = DeepSeek, Tool 2 = ChatGPT, and Tool 3 = Gemini. Higher scores indicate better quality.

**Figure 3:**
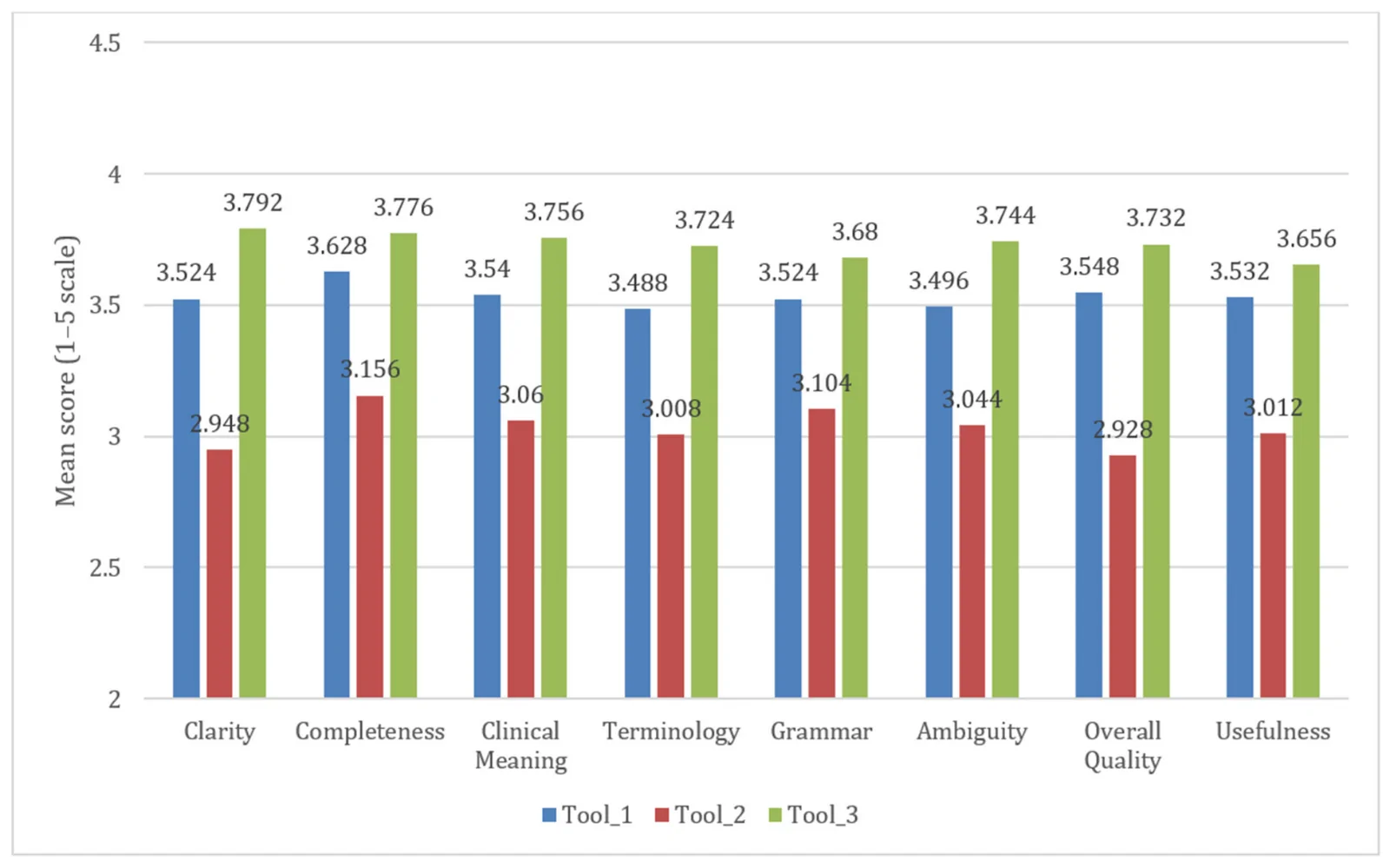
Mean scores across eight translation quality domains for DeepSeek, ChatGPT, and Gemini. Higher scores indicate better performance on the 1–5 scale.

**Figure 4:**
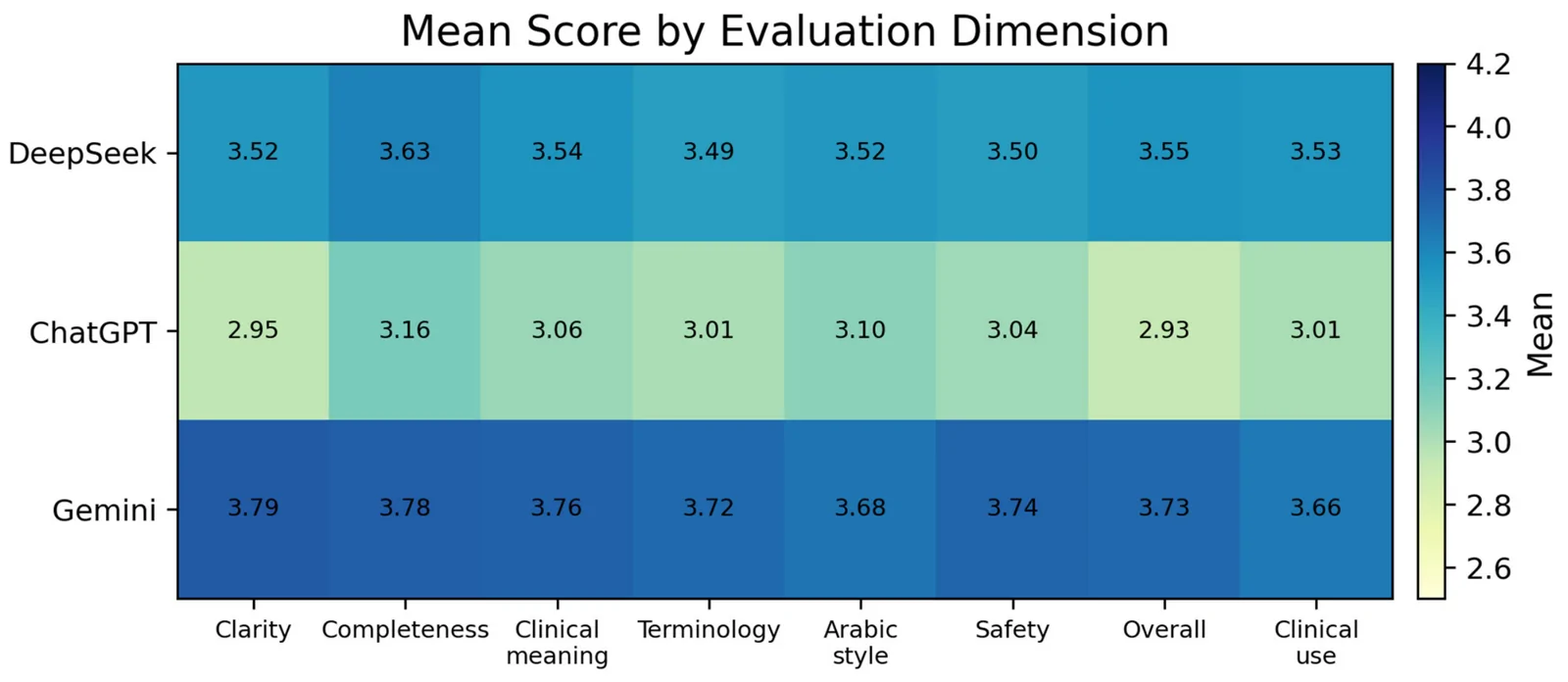
A heatmap of mean scores for each LLM across the eight evaluation domains. Darker cells indicate higher mean scores.

Descriptive statistics are reported as the mean ± standard deviation (SD). For each evaluator, repeated ratings for the three tools were compared using the Friedman test. Kendall’s W was calculated as an effect size measure. When the Friedman test was significant, paired Wilcoxon signed-rank tests were used for post hoc comparisons with multiplicity correction. Statistical significance was set at *p* < 0.05 after adjustment and confidence intervals were calculated at the 95% level. Tool labels were decoded after completion of the analyses. Domain-level results and pairwise comparisons are presented in Table 2, Table 3 and Table 4 and Figure 2, Figure 3 and Figure 4. In addition, laterality accuracy (correct designation of right versus left breast, including ipsilateral references) was verified across all translated outputs by cross-checking against the original source reports. We also conducted an exploratory subgroup comparison of overall and domain-level ratings by evaluator years of experience (≤5 years vs. >5 years) using Mann–Whitney U tests.

To address the hierarchical structure of the data, a supplementary linear mixed-effects model was fit on the full unaggregated rating dataset. Tool, domain, and their interaction were specified as fixed effects; evaluator and report were specified as crossed random intercepts (variance components structure), estimated via restricted maximum likelihood. Estimated marginal means for tool were compared using Bonferroni-adjusted pairwise contrasts with 95% confidence intervals.

Internal consistency across the eight rating domains was high for all three models (Cronbach’s α = 0.983 for DeepSeek, 0.979 for ChatGPT, and 0.989 for Gemini), with inter-item correlations above 0.90. This indicates substantial conceptual overlap among the evaluated domains, suggesting that they largely reflect a single underlying construct of perceived translation quality rather than fully independent dimensions.

## 3. Results

### 3.1. Evaluator Characteristics

Fifty radiologists completed the evaluation (Table 1). The largest age group was 25–34 years (42.0%), followed by 35–44 years (32.0%). Twenty-four evaluators (48.0%) were consultant radiologists, and twenty (40.0%) were residents or registrars. Most participants had at least intermediate English proficiency (92.0%), while 49 (98.0%) were native Arabic speakers. Thirty-two participants (64.0%) reported moderate or high trust in AI for medical translation; however, twenty-nine (58.0%) had not previously used AI for translation.

### 3.2. Overall Model Comparison

Gemini received the highest overall mean score (3.73 ± 0.78), followed by DeepSeek (3.54 ± 0.73) and ChatGPT (3.03 ± 0.70) (Table 4; Figure 2). The proportion of evaluations with a high-quality average score (mean ≥ 4) was 40.0% for Gemini, 34.0% for DeepSeek, and 6.0% for ChatGPT. The overall Friedman test showed a statistically significant difference among tools (χ^2^ = 34.11, df = 2, *p* < 0.001), with a moderate effect size (Kendall’s W = 0.341). In overall pairwise comparisons, DeepSeek outperformed ChatGPT (adjusted *p* < 0.001), Gemini outperformed ChatGPT (adjusted *p* < 0.001), and Gemini also outperformed DeepSeek (adjusted *p* = 0.009).

To account for the hierarchical structure of the data, a linear mixed-effects model was fit on the full unaggregated ratings, with tool, domain, and their interaction as fixed effects, and evaluator and report specified as crossed random intercepts. The model confirmed a significant effect of tool (F(2,5923) = 455.60, *p* < 0.001) and a marginal effect of domain (F(7,5923) = 2.02, *p* = 0.048), with no significant tool-by-domain interaction (F(14,5923) = 1.11, *p* = 0.342). The random-effect variance for evaluator was substantial and significant (variance = 0.374, SE = 0.077, *p* < 0.001), whereas the variance attributable to report was small and not statistically significant (variance = 0.005, SE = 0.004, *p* = 0.198), indicating that the five evaluated reports did not differ systematically once evaluator-level clustering was accounted for. Bonferroni-adjusted pairwise comparisons of estimated marginal means confirmed the primary analysis: Gemini outperformed both DeepSeek (mean difference = 0.197, 95% CI [0.140, 0.255], *p* < 0.001) and ChatGPT (mean difference = 0.699, 95% CI [0.643, 0.757], *p* < 0.001), and DeepSeek outperformed ChatGPT (mean difference = 0.502, 95% CI [0.445, 0.560], *p* < 0.001).

### 3.3. Domain-Level Performance

The same rank order was observed across all eight evaluated domains: Gemini ranked highest, DeepSeek ranked second, and ChatGPT ranked lowest (Table 2; Figure 3). Gemini’s domain scores ranged from 3.66 for clinical communication usefulness to 3.79 for clarity/readability. DeepSeek’s scores ranged from 3.49 for medical terminology to 3.63 for completeness, whereas ChatGPT’s scores ranged from 2.93 for overall quality to 3.16 for completeness.

Friedman tests were significant for every domain (all *p* < 0.001; Table 3). The difference between Gemini and ChatGPT was significant across all domains after multiplicity correction. DeepSeek also outperformed ChatGPT in every domain. Although Gemini had a numerically higher mean score than DeepSeek in all domains, their difference was not statistically significant for completeness, grammar/style, overall quality, or usefulness for clinical communication after the reported correction (Table 4).

### 3.4. Laterality Accuracy

The structured review of all 15 translations (5 reports × 3 tools) confirmed no laterality errors. Right and left breast designations, including ipsilateral references and bilateral/multifocal findings in Reports 4 and 5, were preserved correctly by DeepSeek, ChatGPT, and Gemini in every instance.

### 3.5. Evaluator Subgroup Comparison by Experience

Exploratory analysis by years of experience found no significant differences in ratings of DeepSeek or Gemini across any of the eight evaluated domains. Evaluators with ≤5 years of experience rated ChatGPT significantly higher than those with >5 years of experience for overall quality (U = 169.5, Z = −2.67, *p* = 0.008), grammar/style (U = 199.5, Z = −2.08, *p* = 0.038), and usefulness for clinical communication (U = 193.5, Z = −2.20, *p* = 0.028). Given the small subgroup sizes of evaluators (*n* = 21 and *n* = 29), these findings should be interpreted with caution.

## 4. Discussion

This blinded expert evaluation study found that patient-friendly Arabic translations of breast imaging reports differed meaningfully by LLM despite the use of the same structured prompt. Gemini achieved the highest overall score and the highest score in every evaluated domain, DeepSeek ranked second, and ChatGPT ranked lowest. The domain-level pattern is clinically important because it extends beyond linguistic fluency to completeness, the preservation of clinical meaning, terminology, the safety of wording, and usefulness for clinical communication.

Although mean score differences between models ranged from approximately 0.2 to 0.7 points on the 5-point scale, their clinical relevance should be interpreted alongside the categorical distribution of ratings rather than mean differences alone. The proportion of evaluations rated as high-quality (mean score ≥ 4) differed substantially between models: 40.0% for Gemini, 34.0% for DeepSeek, and only 6.0% for ChatGPT, indicating that model differences reflect meaningfully different likelihoods of producing translations that experts consistently rated higher for clinical communication quality, the preservation of clinical meaning, and safety-related characteristics. The moderate overall effect size (Kendall’s W = 0.341) further supports that these differences are consistent rather than attributable to noise. Smaller domain-level differences, such as those between Gemini and DeepSeek, may carry less practical significance despite statistical significance and should not be over-interpreted as indicating a clinically meaningful safety gap between these two tools.

Our findings are consistent with the broader evidence that LLM-generated report explanations can make radiology information easier for patients to understand, although readability gains do not guarantee clinical safety [3,4,5,18,19]. In a randomized study of knee MRI reports, AI-translated reports improved quiz-based comprehension and were preferred for clarity, but participants expressed slightly lower trust in the AI version than in the original radiologist report [19]. Likewise, a recent randomized study of MRI fistula summaries found better patient-rated readability, comprehension, and usefulness, yet clinician review still identified hallucinations and unverified recommendations [18]. These observations help explain why the present study assessed clinical meaning, medical terminology, and safety separately from readability, rather than treating a fluent Arabic output as sufficient evidence of quality [3,5,20].

The perspectives of radiology professionals and patients should be viewed as complementary rather than interchangeable. Our previous systematic review demonstrated that patients consistently expressed a desire to access radiology reports, while concerns related to comprehension and anxiety remained important. The review also identified considerably fewer studies examining the opinions of radiologists and referring physicians and recommended a more balanced representation of these professional stakeholders. The present study contributes specifically to this gap by examining radiology professionals’ opinions of LLM-generated patient-friendly Arabic translations. Direct evaluation by Arabic-speaking patients remains a separate and necessary subsequent stage of research [21].

The model-specific ranking in our study also mirrors comparative work showing that LLMs have distinct communication profiles under comparable task conditions. In a 100-report expert evaluation, ChatGPT, Gemini, and Copilot all achieved high medical correctness scores, but differed in readability, patient guidance, uncertainty language, and clinical suggestions, supporting context-specific model selection rather than a one-model-fits-all approach [22]. In a comparative study of simplified interventional radiology reports, GPT-4 and Claude-3-Opus generally performed best, but all evaluated models produced some content, coherence, or trust-breaking errors [20]. Similarly, GPT-4 and DeepSeek generated breast cancer structured reports with comparable overall extraction accuracy but showed model-specific weaknesses for particular metastatic sites, illustrating that strong aggregate performance may conceal errors in clinically important subcomponents [23]. This model-specific variability extends to BI-RADS knowledge itself. In a direct evaluation of ten LLMs on BI-RADS Atlas text and visual questions, accuracy varied significantly by category, and models showed category-specific failure points; for example, Gemini 1.5 Pro answered no BI-RADS 5 questions correctly, while ChatGPT 4V failed on all BI-RADS 1 questions [24].

Breast imaging is a particularly demanding setting for patient-facing translation because its value depends on retaining a tightly linked set of structured descriptors and management information. BI-RADS provides standardized terminology, assessment categories, and follow-up guidance across mammography, ultrasound, MRI, and digital breast tomosynthesis; so, changes to category labels or recommendations can alter the practical meaning of a report [8]. Breast report studies have also shown that clinically useful AI processing requires attention to details such as lesion location, size, breast density, imaging modality, BI-RADS category, and diagnostic suggestions rather than broad text similarity alone [7,23,25]. The inclusion of BI-RADS categories 0, 2, 3, 4, and 6 in our study therefore tested not only benign versus malignant language, but also incomplete assessment, short-interval surveillance, biopsy-related communication, and known malignancy scenarios with different risks of patient misunderstanding [8].

Our results also add Arabic-specific evidence to the literature in which multilingual translation performance remains heterogeneous. In a two-center evaluation of 400 GPT-4o translations, factual correctness and completeness varied significantly across target languages, and potentially harmful errors occurred despite generally favorable readability ratings [12]. Meddeb and colleagues similarly found that no single LLM performed best across all language pairs and noted that terminology fidelity required particular scrutiny in multilingual radiology translation [14]. In Hindi report translation, Gupta et al. found that both the selected LLM and prompt wording affected translation metrics, while radiologist review still identified misinterpretations and omissions [13]. A recent narrative review also identified mixed accuracy and nuance-related issues for Arabic radiology translations, emphasizing the need for bilingual clinical review rather than assuming that results from high-resource language pairs generalize to Arabic [6].

Prompt engineering is an important risk reduction strategy, but it cannot make LLM output deterministic or clinically infallible. A study showed that prompt learning could improve the quality and factual retention of plain-language radiology report translations, particularly with GPT-4 [16]. However, prompt effects can be mixed: in the interventional radiology study, few-shot prompting improved some GPT-3.5 measures such as completeness and clinical relevance but was associated with poorer clarity and organization [20]. A recent scoping review of LLM use on radiology reports found that prompt details were often reported, whereas explicit management of output stochasticity was much less common, limiting reproducibility across studies [15]. By using one structured prompt for all three models, our study isolated model-related variation; future work should additionally report model versions, dates of access, parameter settings, repeated-query procedures, and any output selection rules [15,26]. However, this design does not assess prompt sensitivity, and different prompt formulations may substantially alter model outputs and relative performance rankings; the findings reported here should be interpreted as reflecting model performance under one specific, clinically oriented prompt design rather than each model’s full achievable range.

The present findings also support a more rigorous evaluation framework for patient-facing breast report translation. Prior reviews have highlighted substantial heterogeneity in report inputs, model configurations, rater types, readability measures, and safety definitions, which limits direct comparison across studies [4,5,15]. Breast imaging offers an opportunity to supplement subjective ratings with automated verification of safety-critical fields, including right-versus-left laterality, lesion measurements, units, breast density categories, BI-RADS labels, and follow-up recommendations [8,25,27]. In this study, laterality accuracy was verified across all translated outputs, and no errors were identified in right/left breast designation for any of the three models. Such checks would not replace expert review, but they could identify high-risk deviations before a translation reaches a patient. Future studies should develop larger multilingual benchmark sets, use diverse report styles and imaging modalities, involve both patients and bilingual clinicians, and test external datasets because current radiology NLP research commonly shows performance decreases outside the original data environment [25,26].

Finally, our results favor a clinician-mediated implementation model rather than autonomous patient release. Generative AI in radiology has clear potential for patient communication, but published guidance consistently emphasizes ongoing professional oversight, awareness of bias, and secure governance for clinical translation [6,28]. Implementation risk differs by category: a translation error in BI-RADS 3 language could be read as clearing the patient rather than as a surveillance instruction, risking a missed follow-up; an error in BI-RADS 4 language could understate suspicion and delay biopsy; and an error in BI-RADS 6 language could miscommunicate an already-confirmed diagnosis at a psychologically critical stage, adding unwarranted distress or false hope. These category-specific failure modes reinforce that translation quality alone does not guarantee safe deployment without radiologist review.

This caution is reinforced by evidence showing that ChatGPT can use confident language even when incorrect on radiology classification and calculation tasks and by breast cancer decision support research showing that LLMs cannot fully reproduce the complex judgment developed through clinical experience [29,30]. A practical workflow would use de-identified report text within an approved environment, a validated structured prompt, automated checks for preserved report elements, and radiologist review before a patient-friendly Arabic version is released. The next research step should determine whether such radiologist-reviewed translations improve comprehension, confidence, follow-up behavior, anxiety, and trust among Arabic-speaking patients in real breast imaging care settings [5,17,18,27].

### Strengths, Limitations, and Future Work

A major strength was the use of the same structured prompt across three models, allowing for a comparison of model behavior while holding the instruction set constant. The blinded design, inclusion of radiologists with Arabic-language expertise, and evaluation of eight clinical and linguistic domains strengthened the assessment. The case set intentionally covered clinically distinct BI-RADS categories and multiple breast imaging modalities. In addition, this study used a single translation output per model per report, without repeated generations across sessions. LLM outputs may vary across repeated runs even when the prompt and model parameters remain unchanged [31].

One limitation of this study was the use of five reports, one per BI-RADS category; while sufficient to span the clinically distinct categories evaluated, this does not capture the full linguistic and technical variability of real-world breast imaging reports (e.g., dictation styles, report lengths, institutional templates, less common terminology), nor the within-category heterogeneity of categories such as BI-RADS 4, which spans a range of suspicion subtiers (4A–4C) and management recommendations that a single report cannot represent. Statistical power derived from the 50-evaluator repeated-measures design, rather than report-level replication, and a retrospective power analysis confirmed that the evaluator sample exceeded the minimum required for the primary comparison (*N* = 28). The report, rather than the evaluator, remains the principal unit determining the generalizability of these findings. Findings at the category level should therefore be interpreted as evidence from representative exemplars rather than as fully generalizable to all reports within a given category. Future work should expand the report set across multiple institutions, reporting styles, and category subtiers to test generalizability beyond the case types evaluated here.

Another limitation of this study is that all participating radiologists were based in Saudi Arabia. Although Saudi Arabia is a major Arabic-speaking setting where Arabic is the first language, terminology preferences, dialectal variation, clinical communication practices, and healthcare contexts may differ across other Arab countries. Future studies should therefore include radiologists and patients from multiple Arabic-speaking countries to assess broader regional applicability.

## 5. Conclusions

LLMs may help make breast imaging reports more accessible to Arabic-speaking patients, but their performance is model-dependent. As LLMs continue to evolve rapidly, this ranking reflects a snapshot at the time of evaluation and may not hold for future model versions. In this blinded expert evaluation, Gemini achieved the highest ratings, DeepSeek was competitive, and ChatGPT ranked lower across all assessed domains. Because translation errors involving laterality, measurements, BI-RADS labels, lesion location, or recommendations can change clinical understanding, Arabic LLM translations should be implemented only as radiologist-reviewed communication aids. Further patient-centered and real-world validation is needed before routine clinical deployment.

## Figures and Tables

**Table 1:** Participant demographic characteristics and AI translation experience among 50 radiology evaluators.

Characteristic	Category	*n* (%)
Age Group	25–34 years	21 (42%)
	35–44 years	16 (32%)
	45–54 years	12 (24%)
	55–64 years	1 (2%)
Gender	Male	28 (56%)
	Female	22 (44%)
Years in Radiology Practice	0–2	6 (12%)
	3–5	15 (30%)
	6–10	4 (8%)
	11–15	8 (16%)
	16–20	6 (12%)
	21+	11 (22%)
Native Language	Arabic	49 (98%)
	English	1 (2%)
English Proficiency	Basic	4 (8%)
	Intermediate	15 (30%)
	Advanced	31 (62%)
Professional Role	Radiology resident	14 (28%)
	Radiology registrar	6 (12%)
	Radiology fellow, other specialties	4 (8%)
	Radiology fellow, breast/women’s imaging	2 (4%)
	Consultant radiologist, other specialties	21 (42%)
	Consultant radiologist, breast/women’s imaging	3 (6%)
Used AI for Translation	No	29 (58%)
	Unsure	3 (6%)
	Yes	18 (36%)
Trust in AI for Medical Translation	No trust at all	3 (6%)
	Low trust	10 (20%)
	Moderate trust	31 (62%)
	High trust	6 (12%)

**Table 2:** Mean (SD) ratings by domain and model on the 1–5 scale.

Domain	DeepSeek	ChatGPT	Gemini
Clarity/readability	3.52 ± 0.75	2.95 ± 0.71	3.79 ± 0.79
Completeness	3.63 ± 0.80	3.16 ± 0.77	3.78 ± 0.88
Clinical meaning	3.54 ± 0.78	3.06 ± 0.72	3.76 ± 0.84
Medical terminology	3.49 ± 0.74	3.01 ± 0.72	3.72 ± 0.82
Arabic grammar/style	3.52 ± 0.78	3.10 ± 0.78	3.68 ± 0.82
Clinical safety/ambiguity	3.50 ± 0.80	3.04 ± 0.72	3.74 ± 0.79
Overall quality	3.55 ± 0.76	2.93 ± 0.73	3.73 ± 0.77
Clinical communication usefulness	3.53 ± 0.77	3.01 ± 0.81	3.66 ± 0.78

**Table 3:** Friedman tests comparing the three blinded tools across the eight evaluation domains (*N* = 50).

Dimension	Tool 1 * Mean Rank	Tool 2 * Mean Rank	Tool 3 * Mean Rank	χ^2^	*p*
Clarity	2.01	1.44	2.55	34.618	<0.001
Completeness	2.07	1.44	2.49	32.859	<0.001
Clinical Meaning	2.03	1.47	2.50	30.563	<0.001
Terminology	2.00	1.44	2.56	35.636	<0.001
Grammar	2.04	1.57	2.39	20.646	<0.001
Ambiguity	1.98	1.48	2.54	33.476	<0.001
Overall Quality	2.07	1.39	2.54	37.562	<0.001
Usefulness	2.06	1.47	2.47	30.817	<0.001

* Tool 1 = DeepSeek; Tool 2 = ChatGPT; Tool 3 = Gemini. df = degrees of freedom (= 2 for all tests). Higher mean ranks indicate better performance.

**Table 4:** Pairwise post hoc comparison summary after multiplicity correction.

Comparison	Significant Domains	Not Significant Domains
DeepSeek vs. ChatGPT	All 8 domains (*p* < 0.001)	None
Gemini vs. ChatGPT	All 8 domains (*p* < 0.001)	None
DeepSeek vs. Gemini	Clarity, clinical meaning, terminology, clinical safety/ambiguity	Completeness, grammar/style, overall quality, usefulness

## Data Availability

The data that support the findings of this study are available from the corresponding author upon reasonable request.

## Supplementary Materials

The following supporting information can be downloaded at https://www.mdpi.com/article/10.3390/diagnostics16162495/s1: Supplementary Material S1: English Source Breast Imaging Reports and Corresponding LLM-Generated Arabic Translations; Supplementary Material S2: Characteristics of five source breast imaging reports used for LLM translation evaluation; Supplementary Material S3: Prompt.
