# Supplementary material for: Large Language Model Translation of BI-RADS Breast Imaging Reports into Arabic: A Blinded Expert Evaluation of Diagnostic Communication Safety
Source: Diagnostics (Basel). 2026 Aug 7;16(16):2495. doi: 10.3390/diagnostics16162495 (PMC13510894; doi:10.3390/diagnostics16162495)
Supplement: Supplementary file 1 [file diagnostics-16-02495-s001.zip › Supplementary Material S1.pdf]

Supplementary Material 1: English-Source Breast Imaging Reports and Corresponding LLM-Generated Arabic Translations

DeepSeek

Report Number # 1

MRN 8100031284. DATE: June 27, 2021
MODALITY: MAMMOGRAM—DIAGNOSTIC & ULTRASOUND BREAST—BILATERAL:
CLINICAL INDICATION:
Right breast nodule seen on recent CT scan.

FINDINGS ON MAMMOGRAM:
Heterogeneous fibroglandular densities may obscure small pathology.
There is focal asymmetry in the right breast around the 6 o'clock axis. It is partially included due to the deep location and overlaps a circumscribed oval nodule seen in exaggerated medial CC view. No associated microcalcifications in the provided images. It partially persisted on spot compression view; however, evaluation remains suboptimal due to the posterior location of the finding. No nipple retraction or suspicious skin thickening.
On the left side, there are multiple mildly enlarged axillary lymph nodes with mild stranding of the surrounding fat plane. The patient gave a history of recent COVID-19 vaccination taken in the left arm.

FINDINGS ON ULTRASOUND:
Circumscribed oval hypoechoic nodule in right breast at 12 o'clock measures 1.0 x 0.5 x 1.4 cm; it likely corresponds to the mass described on CT scan.
Smaller likely similar nodule noted in right breast at 8 o'clock.
No suspicious masses or abnormality correlates with the focal asymmetry at 6 o'clock.
Abnormal appearance of left axillary lymph nodes with mild uniform borderline cortical thickening likely reactive.
IMPRESSION:
Non-palpable benign-looking right breast mass at 12 o'clock likely corresponds to the finding on CT scan and follow-up recommended.
Partially included deeply located focal asymmetry in the right breast close to the 6 o'clock axis without corresponding suspicious masses on ultrasound; the findings may represent focal asymmetric glandular. Differential diagnosis includes radial scar or less likely malignancy; further evaluation by MRI is recommended.

BI-RADS 0.

"رقم التقرير # 1

الرقم الطبي: 8100031284 – التاريخ: 27 يونيو 2021

طريقة الفحص: تصوير الثدي الشعاعي (تشخيصي) وتصوير الثدي بالموجات فوق الصوتية – للجانبين.

الاستطباب السريري: وجود كتلة في الثدي الأيمن ظهرت في تصوير مقطعي حديث.

نتائج تصوير الثدي الشعاعي (الماموجرام:(
存在توجد كثافات غدية ليفية غير متجانسة قد تحجب رؤية التغيرات الصغيرة.
توجد عدم تناسق بؤري في الثدي الأيمن في الجزء السفلي. يظهر هذا الجزء بشكل غير كامل نظرًا لوقوعه في عمق الثدي، ويتداخل مع كتلة بيضاوية محددة الحواف تظهر بوضوح في المسقطة الإنسية. لا توجد تكلسات دقيقة مصاحبة في الصور المتوفرة. لقد ظل هذا التباين مرئيًا بشكل جزئي عند استخدام المسقطة الموضعية، ولكن تقييمه لا يزال غير مثالي بسبب موقعه العميق. لا يوجد تراجع في الحلمة أو زيادة سمك مشبوه في الجلد.
في الجانب الأيسر، توجد عدة عقد لمفاوية متضخمة قليلاً في الإبط مع وجود خطوط دهنية خفيفة حولها. أفادت المريضة بأنها تلقت تطعيمًا حديثًا ضد كوفيد-19 في الذراع اليسرى.

نتائج تصوير الثدي بالموجات فوق الصوتية (الألتراساوند:(
توجد كتلة بيضاوية محددة الحواف وغير ناقصة للصدى في الجزء العلوي من الثدي الأيمن، يبلغ قياسها 1.0 × 0.5 × 1.4 سم، ومن المرجح أن تكون مطابقة للكتلة الموصوفة في التصوير المقطعي.
توجد كتلة أخرى أصغر حجمًا ويُحتمل أن تكون مشابهة في الجزء الداخلي من الثدي الأيمن.
لا توجد كتل أو تغيرات مشبوهة تتطابق مع منطقة عدم التماثل البؤري في الجزء السفلي من الثدي الأيمن.
يوجد تضخم في العقد اللمفاوية بالجهة اليسرى مع زيادة طفيفة منتظمة في سمك القشرة، وهذا التغير يبدو تفاعليًا (غير ورمي.(

الخلاصة:
كتلة حميدة المظهر في الثدي الأيمن في الجزء العلوي (غير محسوسة باليد)، ومن المرجح أن تكون مطابقة لما ظهر في التصوير المقطعي، ويُنصح بالمتابعة.
منطقة عدم تماثل بؤرية عميقة في الجزء السفلي من الثدي الأيمن، تظهر بشكل غير كامل في التصوير الشعاعي ولا يقابلها كتل مشبوهة في الموجات فوق الصوتية؛ قد تمثل هذه النتائج نسيجًا غديًا غير متماثل. التشخيصات المحتملة تشمل الندبة الشعاعية أو (بدرجة أقل احتمالاً) ورم خبيث. يُنصح بإجراء تقييم إضافي باستخدام التصوير بالرنين المغناطيسي.

**تصنيف) 0 **:BI-RADS غير مكتمل — يحتاج تصويرًا إضافيًا للتقييم و/أو صورًا سابقة للمقارنة حسب الفحص"(

Report Number # 2

Follow-up of former patient in March 21, 2023
Mammogram diagnostic

CLINICAL DATA:
LCIS post excision for mammogram

FINDINGS:

Study compared to prior mammogram performed in June 2021.
Heterogenous fibroglandular densities.
Status post excision of focal asymmetry in right breast associated with distortion.
Mild postoperative distortion noted on Tomosynthesis.
No developing masses or suspicious microcalcifications.
No nipple retraction or suspicious skin thickening.
Stable appearance of the axillary lymph nodes.

IMPRESSION:
Status post excision of the right-side radial sclerosing pathology with lobular neoplasia.
For risk-appropriate screening.
BI-RADS 2

تقرير رقم ٢

متابعة لمريضة سابقة في ٢١ مارس ٢٠٢٣
تصوير الثدي الإشعاعي التشخيصي

البيانات السريرية:
متابعة بعد استئصال ورم الفصيصات الورمي (LCIS) لمريضة خضعت سابقًا لتصوير الثدي الإشعاعي.

النتائج:
تمت مقارنة الدراسة بفحص سابق للثدي بالأشعة (ماموغرام) أُجري في يونيو ٢٠٢١.
أنسجة الثدي ذات كثافة ليفية غدية غير متجانسة.
حالة بعد استئصال منطقة من عدم التماثل مصحوبة باعوجاج (تغيّر في النسيج) في الثدي الأيمن.
وجود تغيّر طفيف بعد العملية (اعوجاج) لوحظ باستخدام تقنية التصوير المقطعي للثدي (توموسينثيسيس.(
لا توجد كتل جديدة أو تكلسات دقيقة يشتبه بها.
لا يوجد انكماش في الحلمة أو زيادة سمك مشبوه في الجلد.
تظهر العقد الليمفاوية في الإبط بحالة مستقرة.

الخلاصة:
حالة بعد استئصال نسيج من الجهة اليمنى لمرض تصلّس شعاعي (radial sclerosing pathology) مصحوب بورم الفصيصات الورمي.(lobular neoplasia)
ينصح بإجراء فحوصات دورية مناسبة لمستوى الخطورة.
(BI-RADS 2 حميد)

Report Number # 3

MRN 72591589. DATE: Nov, 11 2025

Ultrasound breast

INDICATIONS:
Breast cancer on hormonal therapy; last mammogram showed BI-RADS 3.

FINDINGS:
There is a circumscribed oval mass in left breast at 6:00, 2 cm from nipple; the nonseparable tiny smaller similar masses/cysts measure collectively 1.3 x 0.3 cm. No significant interval change as compared to ultrasound performed in May 2025 and November 2023.
Circumscribed oval heterogenous isoechoic area/mass at 6:00, 5 cm from nipple, measures 1.8 x 0.6 cm without significant change. It likely represents focal prominent fat tissue rather than genuine lesion. Smaller similar area noticed laterally.
No associated signs of invasion or enlarged lymph nodes.
Right-side mastectomy with signs of axillary dissection.

IMPRESSION:
Stable small mass at 6:00 for more than 2 years.
The additional findings in the same region indicate likely prominent fat lobules rather genuine lesion.
Follow-up by ultrasound after 6 months is recommended [patient will be due for annual mammographic assessment in May 2026].

BI-RADS 3

رقم التقرير # 3

الرقم الطبي 72591589 التاريخ: 11 تشرين الثاني (نوفمبر) 2025

تصوير الثدي بالموجات فوق الصوتية

الاستطبابات: سرطان الثدي، تخضع للعلاج الهرموني، أظهر آخر تصوير شعاعي للثدي نتيجة BI-RADS 3.

النتائج: توجد كتلة بيضاوية محددة الحواف في الثدي الأيسر في الجزء السفلي، على بعد 2 سم من الحلمة، مع كتل/أكياس أصغر مشابهة غير منفصلة عنها، يبلغ قياسها مجتمعة 1.3 × 0.3 سم. لا يوجد تغير يُذكر خلال الفترة منذ آخر تصوير بالموجات فوق الصوتية في مايو 2025 ونوفمبر 2023.
منطقة/كتلة بيضاوية محددة الحواف، غير متجانسة، متساوية الصدى، في الجزء السفلي، على بعد 5 سم من الحلمة، يبلغ قياسها 1.8 × 0.6 سم، دون تغير يُذكر. من المرجح أن تمثل نسيجًا دهنيًا بارزًا موضعيًا وليس آفة حقيقية. تُلاحظ منطقة أصغر مماثلة في الجانب الخارجي.
لا توجد علامات مصاحبة للغزو أو تضخم في العقد الليمفاوية.
تم استئصال الثدي الأيمن مع وجود علامات تشريح الإبط.

الانطباع: كتلة صغيرة مستقرة في الجزء السفلي لأكثر من عامين. النتائج الإضافية في نفس المنطقة هي على الأرجح فصيصات دهنية بارزة وليست آفة حقيقية.

التوصيات: يوصى بالمتابعة بالتصوير بالموجات فوق الصوتية بعد 6 أشهر (ستكون المريضة مستحقة للتقييم السنوي بالتصوير الشعاعي للثدي في مايو 2026).

(غالبًا حميد — يُنصح عادةً بالمتابعة) BI-RADS 3

Report Number # 4

MRN: 13024229 DATE: Feb 20, 2025
MRI breast
INDICATIONS:
Suspicious Breast Lesion for better assessment and evaluation.
TECHNIQUE:
Multiple sequential multiplanar MRI breast with dynamic post-contrast study.
FINDINGS: There is an irregular mass with irregular margins within the nipple and subareolar region measuring 1.3 x 1.3 x 1.7 cm. It contains a central round signal void area representing the postbiopsy marker. It is heterogenous in signal intensity, predominantly isointense, and demonstrates marked rapid initial enhancement with washout.
Another mass with similar features noticed in the upper outer quadrant of left breast measures 1.2 x 0.8 x 1 cm. It is about 4 cm away from the nipple. An additional small irregular mass with irregular margins was noticed at a lower aspect close to the 6:00 position/central retroareolar region about 5 cm from the nipple [image 85 in post-contrast series] .
Segmental heterogenous nonmass enhancement noticed in the left upper outer quadrant and extending from the retroareolar region and 12:00 position reaching the retromammary prepectoral fascia containing subcentimeter rapidly enhancing masses and duct ectasia worrisome for similar pathology and/or associated in situ carcinoma
On the right side, at the outer aspect close to the 9:00 position, there is a deeply located subcentimeter irregular rapidly enhancing mass measuring 0.5 x 0.5 cm and demonstrating washout enhancement. Multiple small cysts noticed in the same area.
Background heterogenous mammary tissue with the heterogenous mild-to-moderate background parenchymal enhancement.
Bilateral subcentimeter cysts noticed and duct ectasia.
The nipple areolar complex on the right side is unremarkable.
No suspicious axillary lymph nodes.
IMPRESSION:
Mass within the left nipple and subareolar region is highly suggestive of malignancy containing postbiopsy marker.
Additional masses and nonmass enhancements as described in the ipsilateral breast likely similar in nature and/or associated in situ disease.
On the right side, there is a small suspicious mass.
BI-RADS 4

تقرير رقم # 4
MRN: 13024229التاريخ: ٢٠ فبراير ٢٠٢٥**

التصوير بالرنين المغناطيسي للثدي

الفحص:
تصوير الثدي بالرنين المغناطيسي

دواعي الإجراء:
وجود آفة مشبوهة في الثدي لمزيد من التقييم والدراسة

التقنية:
تم إجراء تسلسلات متعددة متعددة المستويات للثدي بالرنين المغناطيسي مع دراسة ديناميكية بعد حقن مادة التباين.

النتائج:
توجد كتلة غير منتظمة الشكل وغير منتظمة الحواف في منطقة الحلمة وخلفها مباشرة (المنطقة خلف الحلمية)، قياسها ١.٣ × ١.٣ × ١.٧ سم. تحتوي هذه الكتلة على منطقة مركزية دائرية من الفراغ في الإشارة تمثل علامة ما بعد أخذ الخزعة.
تظهر الكتلة بكثافة إشارة غير متجانسة، وهي في الغالب متساوية الإشارة مع أنسجة الثدي، وتُظهر تباينًا أوليًا سريعًا ومرتفعًا مع ظاهرة الغسل (التخلص السريع من التباين.(

تُلاحظ كتلة أخرى ذات خصائص مشابهة في الربع الخارجي العلوي من الثدي الأيسر، قياسها ١.٢ × ٠.٨ × ١ سم، وتبعد حوالي ٤ سم عن الحلمة.

كما تُلاحظ كتلة صغيرة إضافية غير منتظمة الشكل وذات حواف غير منتظمة في الجزء السفلي من الثدي قرب المنطقة الوسطى الخلفية للحلمية (أسفل منطقة الحلمة)، وتبعد حوالي ٥ سم عن الحلمة [الصورة ٨٥ في سلسلة ما بعد التباين.[

تُلاحظ منطقة من التباين غير الكتلي غير المتجانس تمتد بشكل قطعي في الربع الخارجي العلوي من الثدي الأيسر، وتمتد من المنطقة خلف الحلمية والجزء العلوي من الثدي (المنطقة العليا) لتصل إلى اللفافة أمام العضلة الصدرية خلف أنسجة الثدي.
تحتوي هذه المنطقة على كتل صغيرة جدًا (أقل من سنتيمتر) سريعة التباين، بالإضافة إلى توسع في القنوات اللبنية، مما يثير القلق من وجود نفس الحالة المرضية و/أو سرطان موضعي (في الأقنية.(

في الجانب الأيمن، وبالقرب من الجزء الخارجي من الثدي (ناحية الإبط)، توجد كتلة صغيرة عميقة غير منتظمة الشكل وأقل من سنتيمتر، قياسها ٠.٥ × ٠.٥ سم، وتظهر ظاهرة الغسل في التباين. كما تُلاحظ عدة أكياس صغيرة في نفس المنطقة.

يوجد نسيج ثديي خلفي غير متجانس مع تباين خلفي غير متجانس خفيف إلى متوسط في الشدة.

تُلاحظ أكياس صغيرة (أقل من سنتيمتر) في كلا الثديين، بالإضافة إلى توسع في القنوات اللبنية.

مركب الحلمة والهالة في الثدي الأيمن يبدو طبيعيًا.

لا توجد عقد لمفاوية إبطية مشبوهة.

الخلاصة:
*كتلة في الثدي الأيسر في منطقة الحلمة وخلفها مباشرة، مرجّح جدًا أن تكون خبيثة، وتحتوي على علامة ما بعد الخزعة.
*كتل إضافية ومناطق تباين غير كتلي في نفس الثدي (الأيسر)، يُرجح أن تكون بنفس الطبيعة الخبيثة و/أو مرتبطة بمرض سرطاني موضعي (في الأقنية.(
*كتلة صغيرة مشبوهة في الجانب الأيمن.

تصنيف BI-RADS 4: (اشتباه متوسط للخباثة)

Report Number # 5

MRN: Missed. DATE: April 7, 2025
MRI OF THE BREAST
CLINICAL INDICATION:
A 45-year-old female patient diagnosed with left breast cancer, requires better assessment and evaluation.
Additional history:
Patient has multiple ultrasound-guided biopsies as follows:
1. Right breast, mass at 3 o'clock, core needle biopsy:
- Fibroadenoma.
2. Left breast, mass at 6 o'clock, core needle biopsy:
- Invasive ductal carcinoma of no special type, mSBR grade 3 (tubular formation 3, nuclear pleomorphism 3, mitotic rate 2).
3. Left breast, mass at 8 o'clock, core needle biopsy:
- Benign breast tissue.

TECHNIQUE:
Multiplanar multisequential MRI of the breasts was performed prior to and following the administration of IV contrast gadolinium utilizing routine tumor protocol.
COMPARISON:
None. Correlation was made with mammogram and ultrasound dated February 26, 2025.
FINDINGS:
Almost entirely fat (ACR type A).
Mild symmetric background parenchymal enhancement.
RIGHT BREAST:
There is a circumscribed oval-shaped minimally enhancing lesion in the right breast central inner quadrant of the anterior third of the gland, measuring 1.3 x 0.8 cm with an internal postbiopsy clip, which corresponds to biopsy-proven fibroadenoma.
No suspicious mass or nonmass enhancement.
No skin thickening or nipple retraction.
LEFT BREAST:
There is a spiculated heterogeneously enhancing mass in the left hip breast lower central quadrant of the mid-third of the gland, measuring 2 x 2.3 x 1.9 cm (TV, AP and craniocaudal) with an internal postbiopsy clip, which corresponds to biopsy-proven malignancy.
Just 4.8 cm posterior and inferior to the index mass, there is another irregular enhancing nodule close to the cell, measuring 0.5 cm, worrisome for multifocal disease (series 16, image #76).
Another suspicious enhancing nodule in the upper inner quadrant of the anterior third of the gland measures 0.5 cm (series 16, image #83).

No suspicious enhancement corresponding to findings on prior ultrasound at 3:00.
Postbiopsy clip in the central inner quadrant without underlying suspicious enhancement which corresponds to biopsy-proven benign breast tissue at 8:00.
Progressive foci of enhancement in the subareolar region, measuring 0.4 cm (series 13, image #58).
No skin thickening or nipple retraction.
Multiple suspicious left axillary lymph nodes mainly involving level 1. The largest measures 1.9 x 1 cm.
No enlarged intramammary lymph nodes.
IMPRESSION:
- Biopsy-proven malignancy in the left breast at 6 o'clock with 2 additional suspicious nodules at 6 o'clock posteriorly and in the upper inner quadrant; for a second look, ultrasound and ultrasound-guided biopsy are required to exclude multicentric disease.
- Clearly metastatic ipsilateral axillary lymph nodes.
BI-RADS: 6, Known Biopsy-Proven Malignancy.

تقرير رقم 5

رقم الملف الطبي: غير موجود
التاريخ: ٧ أبريل ٢٠٢٥

تصوير الرنين المغناطيسي للثدي

السبب السريري:
مريضة تبلغ من العمر ٤٥ عامًا تم تشخيص إصابتها بسرطان الثدي الأيسر، وذلك لتقييم وفحص أفضل للحالة.
تاريخ إضافي:
خضعت المريضة لعدة خزعات بإرشاد الموجات فوق الصوتية على النحو التالي:
١. الثدي الأيمن، كتلة في الجزء العلوي الخارجي، خزعة أساسية بإبرة:
-ورم غدي ليفي.
٢. الثدي الأيسر، كتلة في الجزء السفلي من الثدي، خزعة أساسية بإبرة:
-سرطانة قنوية غازية من النوع غير الخاص، الدرجة ٣ حسب مقياس SBR (تكوين أنابيب ٣، تعدد الأشكال النووي ٣، معدل الانقسام ٢).
٣. الثدي الأيسر، كتلة في الجزء السفلي الخارجي، خزعة أساسية بإبرة:
-نسيج ثديي حميد.

التقنية:
تم إجراء تصوير متعدد المستويات والمتسلسلات للثديين بالرنين المغناطيسي قبل وبعد حقن مادة التباين (الجادولينيوم) عن طريق الوريد، باستخدام البروتوكول الروتيني للأورام.

المقارنة:
لا يوجد. تمت المقارنة مع صور الماموجرام والموجات فوق الصوتية المؤرخة في ٢٦ فبراير ٢٠٢٥.

النتائج:
الثديان دهنيان بشكل شبه كامل (النوع A حسب تصنيف ACR).
تعزيز خلفي متناظر خفيف لحمة الثدي.

الثدي الأيمن:
توجد آفة محددة الشكل بيضاوية ذات تعزيز ضئيل في الربع الداخلي الأوسط من الثدي الأيمن، في الثلث الأمامي من الغدة، حجمها ١.٣ × ٠.٨ سم وتحتوي على مشبك داخلي بعد الخزعة، وهي تتوافق مع الورم الغدي الليفي المؤكد بالخزعة.
لا توجد كتلة مشبوهة أو تعزيز غير كتلي.
لا يوجد زيادة في سمك الجلد أو تراجع في الحلمة.

الثدي الأيسر:
توجد كتلة شائكة غير منتظمة الشكل ذات تعزيز غير متجانس في الربع السفلي الأوسط من الثدي الأيسر، في الثلث الأوسط من الغدة، حجمها ٢ × ٢.٣ × ١.٩ سم (عرضي، أمامي-خلفي، والقحفي-الذنبي) وتحتوي على مشبك داخلي بعد الخزعة، وهي تتوافق مع الورم الخبيث المؤكد بالخزعة.
على بُعد ٤.٨ سم خلف وأسفل الكتلة الأساسية، توجد عقيدة معززة أخرى غير منتظمة بالقرب من جدار الصدر، حجمها ٠.٥ سم، وتثير القلق بشأن مرض متعدد البؤر (السلسلة ١٦، الصورة رقم ٧٦).
عقيدة مشبوهة أخرى معززة في الربع الداخلي العلوي، في الثلث الأمامي من الغدة، حجمها ٠.٥ سم (السلسلة ١٦، الصورة رقم ٨٣).
لا يوجد تعزيز مشبوه يتوافق مع النتائج في التصوير بالموجات فوق الصوتية السابق في الجزء العلوي الخارجي.
يوجد مشبك بعد الخزعة في الربع الداخلي الأوسط بدون تعزيز مشبوه تحته، وهو يتوافق مع النسيج الثديي الحميد المؤكد بالخزعة في الجزء السفلي الخارجي.
بؤ تعزيز متزايدة في المنطقة خلف الحلمة، حجمها ٠.٤ سم (السلسلة ١٣، الصورة رقم ٥٨).
لا يوجد زيادة في سمك الجلد أو تراجع في الحلمة.
توجد عدة عقد لمفاوية إبطية يسرى مشبوهة، تشمل بشكل رئيسي المستوى الأول. أكبرها حجمها ١.٩ × ١ سم.
لا توجد عقد لمفاوية داخل الثدي متضخمة.

الانطباع:
-ورم خبيث مؤكد بالخزعة في الثدي الأيسر في الجزء السفلي من الثدي مع وجود عقيدتين مشبوهتين إضافيتين في الخلف من نفس المنطقة وفي الربع الداخلي العلوي، وذلك لإجراء فحص موجات فوق صوتية ثانوي وخزعة موجّهة بالموجات فوق الصوتية لاستبعاد مرض متعدد المراكز.
-عقد لمفاوية إبطية في نفس الجانب تُظهر بوضوح علامات وجود ورم خبيث منتشر.

تصنيف BI-RADS: ٦ (خباثة مؤكدة بالخزعة)

Gemini

Report Number # 1

MRN 8100031284. DATE June 27, 2021
MODALITY: MAMMOGRAM—DIAGNOSTIC & ULTRASOUND BREAST—BILATERAL:
CLINICAL INDICATION:
Right breast nodule seen on recent CT scan.

FINDINGS ON MAMMOGRAM:
Heterogeneous fibroglandular densities may obscure small pathology.

There is focal asymmetry in the right breast around the 6 o'clock axis. It is partially included due to the deep location and overlaps a circumscribed oval nodule seen in exaggerated medial CC view. No associated microcalcifications in the provided images. It partially persisted on spot compression view; however, evaluation remains suboptimal due to the posterior location of the finding. No nipple retraction or suspicious skin thickening.
On the left side, there are multiple mildly enlarged axillary lymph nodes with mild stranding of the surrounding fat plane. The patient gave a history of recent COVID-19 vaccination taken in the left arm.

FINDINGS ON ULTRASOUND:
A circumscribed oval hypoechoic nodule in the right breast at 12 o'clock measures 1.0 x 0.5 x 1.4 cm; it likely corresponds to the mass described on CT scan.
A smaller likely similar nodule noted in the right breast at 8 o'clock.
No suspicious masses or abnormality correlates with the focal asymmetry at 6 o'clock.
Abnormal appearance of left axillary lymph nodes with mild uniform borderline cortical thickening likely reactive.
IMPRESSION:
Non-palpable benign-looking right breast mass at 12 o'clock likely corresponds to the finding on CT scan and follow-up recommended.
Partially included deeply located focal asymmetry in the right breast close to the 6 o'clock axis without corresponding suspicious masses on ultrasound; the findings may represent focal asymmetric glandular. Differential diagnosis includes radial scar or less likely malignancy; further evaluation by MRI is recommended.

BI-RADS 0.

"بناءً على التعليمات المزودة، إليك ترجمة تقرير الأشعة إلى لغة عربية ميسرة للمريض مع الحفاظ على الدقة الطبية والهيكل الأصلي:

رقم التقرير # 1

MRN 8100031284 - التاريخ: 27 يونيو 2021

ULTRASOUND BREAST - & MODALITY: MAMMOGRAM – DIAGNOSTIC :BILATERAL

(طريقة الفحص: تصوير الثدي بالأشعة السينية (الماموجرام التشخيصي) والموجات فوق الصوتية للثديين)

:CLINICAL INDICATION

(دواعي الفحص السريرية)
كتلة (عقيدة) في الثدي الأيمن شوهدت في فحص بالأشعة المقطعية (CT scan) أجري مؤخراً.

:FINDINGS ON MAMMOGRAM

(النتائج في فحص الماموجرام)
توجد أنسجة غدية ليفية غير متجانسة الكثافة مما قد يحجب الأمراض الصغيرة.
يوجد عدم تماثل بؤري في الثدي الأيمن حول الجزء السفلي من الثدي. تم تضمين هذا الجزء جزئياً في الأشعة نظراً لموقعه العميق، وهو يتداخل مع كتلة (عقيدة) بيضاوية محددة بوضوح تظهر في عرض (exaggerated medial CC view).
لا توجد تكلسات دقيقة مرتبطة بها في الصور المقدمة. استمر هذا الظهور جزئياً في لقطة الضغط الموضعي، ومع ذلك، لا يزال التقييم أقل من المثالي بسبب الموقع الخلفي لهذا الاكتشاف. لا يوجد انكماش في الحلمة أو تسمك جلدي مشبوه.
في الجانب الأيسر، توجد عدة غدد لمفاوية إبطية متضخمة قليلاً مع وجود تجمعات خفيفة في طبقة الدهون المحيطة. ذكرت المريضة تاريخاً لتلقي لقاح كوفيد (COVID) مؤخراً في الذراع اليسرى.

:FINDINGS ON ULTRASOUND
(النتائج في فحص الموجات فوق الصوتية)
كتلة (عقيدة) بيضاوية محددة ومنخفضة الصدى في الثدي الأيمن في الجزء العلوي من الثدي، تبلغ قياساتها 1.0 × 0.5 × 1.4 سم، ومن المرجح أنها تتوافق مع الكتلة الموصوفة في فحص الأشعة المقطعية (CT scan).
لوحظت كتلة (عقيدة) أصغر حجماً ومشابهة على الأرجح في الثدي الأيمن في الجزء السفلي الخارجي (باتجاه الإبط).
لا توجد كتل مشبوهة أو اختلالات ترتبط بعدم التماثل البؤري الموجود في الجزء السفلي من الثدي.
شكل غير طبيعي للغدد اللمفاوية الإبطية اليسرى مع تسمك قشري طفيف ومنتظم في الحدود الدنيا، وهو على الأرجح ناتج عن رد فعل (تفاعلي).
:IMPRESSION
(الانطباع/الخلاصة)
كتلة في الثدي الأيمن غير محسوسة وتبدو حميدة في الجزء العلوي من الثدي، تتوافق على الأرجح مع ما وجد في فحص الأشعة المقطعية (CT scan)، ويُنصح بالمتابعة.
عدم تماثل بؤري يظهر جزئياً ويقع في مكان عميق في الثدي الأيمن بالقرب من الجزء السفلي من الثدي دون وجود كتل مشبوهة مقابلة في فحص الموجات فوق الصوتية؛ قد تمثل هذه النتائج نسيج غدي ليفي بؤري طبيعي، ومع ذلك، يُنصح بإجراء تصوير ماموجرام تشخيصي إضافي و/أو مقارنة مع صور ماموجرام سابقة لزيادة التأكيد.
تغيرات تفاعلية (نتيجة لقاح كوفيد 19 الأخير) في الغدد اللمفاوية الإبطية اليسرى.
RECOMMENDATIONS:
التوصيات
يُنصح بإجراء تصوير إضافي و/أو إحضار أفلام سابقة للمقارنة.
BI-RADS: 0 غير مكتمل – يحتاج لتقييم إضافي بالصور و/أو مقارنة مع ماموجرام سابق

Report Number # 2

Follow up of former patient in March 21, 2023
Mammogram diagnostic

CLINICAL DATA:
LCIS post excision for mammogram

FINDINGS:
Study compared to prior mammogram performed in June 2021
Heterogenous fibroglandular densities
Status post excision of focal asymmetry in right breast associated with distortion.
Mild postoperative distortion noted on Tomosynthesis.
No developing masses or suspicious microcalcifications.
No nipple retraction or suspicious skin thickening.
Stable appearance of the axillary lymph nodes.

IMPRESSION:
Status post excision of the right-side radial sclerosing pathology with lobular neoplasia.
For risk appropriate screening.

BI-RADS 2

رقم التقرير # 2
متابعة مريضة سابقة في 21 مارس 2023
تصوير الثدي بالأشعة السينية (الماموجرام) التشخيصي
CLINICAL DATA:
حالة ما بعد استئصال LCIS (السرطان الفصيصي الموضعي) لإجراء تصوير الماموجرام.
FINDINGS:
تمت مقارنة الدراسة مع تصوير الماموجرام السابق المجرى في يونيو 2021.
كثافات ليفية غدية غير متجانسة.
حالة ما بعد استئصال لعدم تماثل بؤري في الثدي الأيمن مرتبط بتشوه في أنسجة الثدي.
يلاحظ وجود تشوه طفيف ما بعد الجراحة في التصوير المقطعي للثدي (Tomosynthesis).
لا توجد كتل مستجدة أو تكلسات دقيقة مشبوهة.
لا يوجد انكماش في الحلمة أو تسمك مشبوه في الجلد.
مظهر مستقر للعقد الليمفاوية الإبطية.
IMPRESSION:
حالة ما بعد استئصال آفات تليفية شعاعية (radial sclerosing pathology) مع نشأ ورمي فصيصي في الجانب الأيمن.
للمتابعة الدورية المناسبة لمستوى الخطورة.
BI-RADS 2 (حميد)

هل ترغب في أن أشرح لك أي مصطلح طبي ورد في هذا التقرير أو أساعدك في صياغة أسئلة لمناقشتها مع طبيبك؟

Report Number # 3

MRN 72591589. DATE: Nov, 11 2025

Ultrasound breast

INDICATIONS:
Breast cancer on hormonal therapy; last mammogram showed BI-RADS 3.

FINDINGS:
There is a circumscribed oval mass in the left breast at 6:00, 2 cm from nipple, with nonseparable tiny smaller similar masses/cysts measuring collectively 1.3 x 0.3 cm. No significant interval change as compared to ultrasound performed in May 2025 and November 2023.
Circumscribed oval heterogenous isoechoic area/mass at 6:00, 5 cm from nipple measures 1.8 x 0.6 cm without significant change. It likely represents focal prominent fat tissue rather than a genuine lesion. Smaller similar area noticed laterally.
No associated signs of invasion or enlarged lymph nodes.

Right-side mastectomy with signs of axillary dissection.

IMPRESSION:
Stable small mass at 6:00 for more than 2 years.
The additional findings in the same region indicate likely prominent fat lobules rather a genuine lesion.
Follow-up by ultrasound after 6 months is recommended [patient will be due for annual mammographic assessment in May 2026].

BI-RADS 3

تقرير رقم # 3
رقم الملف الطبي: 72591589 التاريخ: 11 نوفمبر 2025
MODALITY (نوع الفحص):
Ultrasound breast (تصوير الثدي بالموجات فوق الصوتية)
INDICATIONS (دواعي الفحص):
سرطان الثدي وتتلقى العلاج الهرموني، أظهر آخر تصوير للماموجرام BI-RADS 3.
FINDINGS (النتائج):
توجد كتلة بيضاوية محددة في الثدي الأيسر في الجزء السفلي من الثدي، على بعد 2 سم من الحلمة، مع وجود كتل صغيرة مماثلة أو أكياس لا يمكن فصلها عنها، تبلغ قياساتها مجتمعة 1.3 × 0.3 سم. لا يوجد تغير ملحوظ مقارنة بفحص الموجات فوق الصوتية الذي أُجري في مايو 2025 ونوفمبر 2023.
منطقة أو كتلة بيضاوية محددة غير متجانسة وذات صدى مماثل للأنسجة المحيطة (isoechoic) في الجزء السفلي من الثدي، على بعد 5 سم من الحلمة، تبلغ قياساتها 1.8 × 0.6 سم دون تغيير ملحوظ. يرجح أنها تمثل نسيجًا دهنيًا بارزًا وليست إصابة حقيقية. لوحظت منطقة مماثلة أصغر حجماً في الجانب الخارجي (باتجاه الإبط).
لا توجد علامات مصاحبة لانتشار أو تضخم في الغدد الليمفاوية.
استئصال الثدي في الجانب الأيمن مع وجود علامات لعملية تنظيف الغدد الإبطية.
IMPRESSION (الانطباع):
كتلة صغيرة مستقرة في الجزء السفلي من الثدي على مدار فترة تزيد عن عامين.
النتائج الإضافية في نفس المنطقة يرجح أنها فصوص دهنية بارزة وليست إصابة حقيقية.
يوصى بالمتابعة عن طريق الموجات فوق الصوتية بعد 6 أشهر [سيكون موعد الفحص السنوي للماموجرام للمريضة في مايو 2026].
BI-RADS 3:
(غالبًا حميد — يُنصح عادةً بالمتابعة)

Next Step: Would you like me to create a summary of the findings specifically highlighting the comparison between this report and the previous ones mentioned?

Report Number # 4

MRN: 13024229. DATE: Feb 20, 2025
MRI breast
INDICATIONS:
Suspicious breast lesion requires better assessment and evaluation.
TECHNIQUE:

Multiple sequential multiplanar MRI breast with dynamic post-contrast study.
FINDINGS: There is an irregular mass with irregular margins within the nipple and subareolar region measuring 1.3 x 1.3 x 1.7 cm. It contains a central round signal void area representing the postbiopsy marker. It is heterogenous in signal intensity, predominantly isointense, and demonstrates marked rapid initial enhancement with washout .
Another mass with similar features noticed in the upper outer quadrant of the left breast measures 1.2 x 0.8 x 1 cm. It is about 4 cm away from the nipple. An additional small irregular mass with irregular margins noticed at a lower aspect close to the 6:00 position/central retroareolar region about 5 cm from the nipple [image 85 in post-contrast series].
Segmental heterogenous nonmass enhancement noticed in the left upper outer quadrant and extending from the retroareolar region and 12:00 position reaching the retromammary prepectoral fascia containing subcentimeter rapidly enhancing masses and duct ectasia, worrisome for similar pathology and/or associated in situ carcinoma.
On the right side, at the outer aspect close to the 9:00 position, there is a deeply located subcentimeter irregular rapidly enhancing mass measuring 0.5 x 0.5 cm and demonstrating washout enhancement. Multiple small cysts noticed in the same area.
Background heterogenous mammary tissue with the heterogenous mild-to-moderate background parenchymal enhancement.
Bilateral subcentimeter cysts noticed and duct ectasia.
The nipple areolar complex on the right side is unremarkable.
No suspicious axillary lymph nodes.
IMPRESSION:
The mass within the left nipple and subareolar region is highly suggestive of malignancy containing postbiopsy marker.
Additional masses and nonmass enhancements as described in the ipsilateral breast likely similar in nature and/or associated in situ disease.
On the right side, there is a small suspicious mass.
BI-RADS 4

رقم التقرير: 4
رقم الملف الطبي: 13024229 التاريخ: 20 فبراير 2025
MODALITY (نوع الفحص):
تصوير الثدي بالرنين المغناطيسي (MRI breast)
INDICATIONS (دواعي الفحص):
آفة مشبوهة بالثدي لمزيد من التقييم والتقدير.
TECHNIQUE (التقنية):
تصوير متعدد المتواليات والمستويات للثدي بالرنين المغناطيسي مع دراسة حركية بعد حقن الصبغة.
FINDINGS (النتائج):
توجد كتلة غير منتظمة ذات حواف غير منتظمة داخل حلمة الثدي ومنطقة ما خلف الهالة (subareolar) بقياس 1.3 × 1.3 × 1.7 سم. تحتوي الكتلة في مركزها على منطقة دائرية خالية من الإشارة تمثل علامة ما بعد الخزعة (postbiopsy marker). إشارة الكتلة غير متجانسة (تتماثل في شدتها مع الأنسجة المحيطة غالبًا) وتظهر تعزيزاً سريعاً وواضحاً للصبغة في البداية مع خاصية التلاشي (washout).
لوحظت كتلة أخرى بخصائص مماثلة في الجزء العلوي الخارجي من الثدي الأيسر بقياس 1.2 × 0.8 × 1 سم، وتبعد حوالي 4 سم عن الحلمة. كما لوحظت كتلة صغيرة إضافية غير منتظمة ذات حواف غير منتظمة في الجزء السفلي قريباً من

منتصف الثدي من الأسفل/المنطقة المركزية خلف الهالة، وتبعد حوالي 5 سم عن الحلمة [صورة رقم 85 في سلسلة ما بعد الصبغة].
لوحظ تعزيز غير كتلي (nonmass enhancement) قطاعي غير متجانس في الجزء العلوي الخارجي من الثدي الأيسر، يمتد من منطقة ما خلف الهالة ومن الجزء العلوي من الثدي (فوق الحلمة) وصولاً إلى اللفافة أمام العضلة الصدرية خلف الثدي، ويحتوي على كتل صغيرة (أقل من سنتيمتر) سريعة التعزيز وتوسع في القنوات الثديية (duct ectasia)، مما يثير القلق من وجود مرض مماثل و/أو سرطان موضعي مرتبط به (in situ carcinoma).
في الجانب الأيمن، وفي الجزء الخارجي (ناحية الإبط) يوجد كتلة عميقة غير منتظمة يقل حجمها عن سنتيمتر، بقياس 0.5 × 0.5 سم، وتظهر تعزيزاً سريعاً للصبغة مع خاصية التلاشي (washout). كما لوحظت أكياس صغيرة متعددة في نفس المنطقة.
خلفية نسيج الثدي غير متجانسة مع تعزيز طفيف إلى متوسط لأنسجة الثدي في الخلفية (background parenchymal enhancement).
لوحظت أكياس ثنائية الجانب يقل حجمها عن سنتيمتر وتوسع في القنوات الثديية.
مركب الحلمة والهالة في الجانب الأيمن طبيعي.
لا توجد غدد ليمفاوية إبطية مشبوهة.

IMPRESSION (الانطباع الطبي):

- كتلة داخل حلمة الثدي الأيسر ومنطقة ما خلف الهالة مرجحة جداً للخباثة وتحتوي على علامة ما بعد الخزعة.
- كتل إضافية وتعزيزات غير كتلية كما هو موضح في نفس الثدي، يُرجح أنها من طبيعة مماثلة و/أو مرتبطة بمرض موضعي (in situ disease).
- كتلة صغيرة مشبوهة في الجانب الأيمن.

BI-RADS:

BI-RADS 4: (اشتباه، يُنصح عادةً بأخذ خزعة للتحقق)

هل ترغب في أن أشرح لك أي مصطلح طبي ورد في هذا التقرير بشكل أكثر تفصيلاً؟

Report Number # 5

MRN: Missed. DATE: April 7, 2025
MRI OF THE BREAST
CLINICAL INDICATION:
A 45-year-old female patient diagnosed with left breast cancer requires better assessment and evaluation.
Additional history:
Patient has multiple ultrasound-guided biopsies as follows:
1. Right breast, mass at 3 o'clock, core needle biopsy:
- Fibroadenoma.
2. Left breast, mass at 6 o’clock, core needle biopsy:
- Invasive ductal carcinoma of no special type, mSBR grade 3 (tubular formation 3, nuclear pleomorphism 3, mitotic rate 2).
3. Left breast, mass at 8 o’clock, core needle biopsy:
- Benign breast tissue.

TECHNIQUE:
Multiplanar multisequential MRI of the breasts was performed prior to and following the administration of IV contrast gadolinium utilizing routine tumor protocol.

COMPARISON:
None. Correlation was made with mammogram and ultrasound dated February 26, 2025.
FINDINGS:
Almost entirely fat (ACR type A).
Mild symmetric background parenchymal enhancement.
RIGHT BREAST:
There is a circumscribed oval-shaped minimally enhancing lesion in the right breast central inner quadrant of the anterior third of the gland, measuring 1.3 x 0.8 cm with an internal postbiopsy clip, which corresponds to biopsy-proven fibroadenoma.
No suspicious mass or nonmass enhancement.
No skin thickening or nipple retraction.
LEFT BREAST:
There is a spiculated heterogeneously enhancing mass in the left hip breast lower central quadrant of the mid-third of the gland, measuring 2 x 2.3 x 1.9 cm (TV, AP and craniocaudal) with an internal postbiopsy clip, which corresponds to biopsy-proven malignancy.
Just 4.8 cm posterior and inferior to the index mass, there is another irregular enhancing nodule close to the cell, measuring 0.5 cm, worrisome for multifocal disease (series 16, image #76).
Another suspicious enhancing nodule in the upper inner quadrant of the anterior third of the gland measures 0.5 cm (series 16, image #83).
No suspicious enhancement corresponding to findings on prior ultrasound at 3:00.
Postbiopsy clip in the central inner quadrant without underlying suspicious enhancement which corresponds to biopsy-proven benign breast tissue at 8:00.
Progressive foci of enhancement in the subareolar region measure 0.4 cm (series 13, image #58).
No skin thickening or nipple retraction.
Multiple suspicious left axillary lymph nodes mainly involving level 1. The largest measures 1.9 x 1 cm.
No enlarged intramammary lymph nodes.
IMPRESSION:
- Biopsy-proven malignancy in the left breast at 6 o'clock with 2 additional suspicious nodules at 6 o'clock posteriorly and in the upper inner quadrant; for a second look, ultrasound and ultrasound-guided biopsy are required to exclude multicentric disease.
- Clearly metastatic ipsilateral axillary lymph nodes.
BI-RADS: 6, Known Biopsy-Proven Malignancy.

رقم التقرير: 5
الرقم الطبي (MRN): غير مذكور. التاريخ: 7 أبريل 2025
MRI OF THE BREAST
تصوير الثدي بالرنين المغناطيسي
CLINICAL INDICATION: (دواعي الفحص السريرية)
مريضة تبلغ من العمر 45 عامًا تم تشخيصها بإصابة بسرطان الثدي الأيسر، وذلك لمزيد من التقييم والتدقيق.
تاريخ طبي إضافي:
خضعت المريضة لعدة خزعات تحت إرشاد الموجات فوق الصوتية (السونار) كما يلي:

1. الثدي الأيمن، كتلة في الجزء الداخلي (جهة منتصف الصدر)، خزعة بالإبرة القاطعة:
- ورم غدي ليفي (Fibroadenoma).
2. الثدي الأيسر، كتلة في الجزء السفلي من الثدي، خزعة بالإبرة القاطعة:
- سرطان قنوي غازي من نوع غير محدد، درجة mSBR 3 (تكون أنبوبي 3، تعدد أشكال النوى 3، معدل الانقسام الفتيلي 2).
3. الثدي الأيسر، كتلة في الجزء السفلي الخارجي (جهة الإبط)، خزعة بالإبرة القاطعة:
- أنسجة ثدي حميدة.

TECHNIQUE: (التقنية)

تم إجراء تصوير بالرنين المغناطيسي للثديين بوضعيات وتسلسلات متعددة قبل وبعد إعطاء صبغة الجادولينيوم الوريدية باتباع البروتوكول الروتيني للأورام.

COMPARISON: (المقارنة)

لا يوجد. تمت المضاهاة مع صور الماموجرام والموجات فوق الصوتية (السونار) المؤرخة في 26 فبراير 2025.

FINDINGS: (النتائج)

الثدي مكون بالكامل تقريبًا من دهون (ACR type A).
تعزيز بسيط ومتماثل في خلفية نسيج الثدي (BPE).

RIGHT BREAST: (الثدي الأيمن)

توجد آفة بيضاوية الشكل محددة بوضوح وذات تعزيز طفيف في الربع المركزي الداخلي للثدي الأيمن في الثلث الأمامي من الغدة، بقياس 1.3 × 0.8 سم مع وجود مشبك معدني داخلي (بعد الخزعة)، وهي تتوافق مع الورم الغدي الليفي المثبت بالخزعة.
لا يوجد تعزيز لكتل مشبوهة أو تعزيز لغير الكتل.
لا يوجد سماكة في الجلد أو تراجع في الحلمة.

LEFT BREAST: (الثدي الأيسر)

توجد كتلة مشوكة وذات تعزيز غير متجانس في الربع المركزي السفلي من الثدي الأيسر في الثلث الأوسط من الغدة، بقياس 2 × 2.3 × 1.9 سم (العرض المستعرض، والقطر الأمامي الخلفي، والقطر الرأسي) مع وجود مشبك معدني داخلي (بعد الخزعة)، وهي تتوافق مع الورم الخبيث المثبت بالخزعة.
على بعد 4.8 سم فقط خلف وأسفل الكتلة الأساسية، توجد عقدة أخرى ذات تعزيز غير منتظم قريبة من الجدار الصدري، بقياس 0.5 سم، وهي مثيرة للقلق لوجود مرض متعدد البؤر (سلسلة 16، صورة رقم 76).
عقدة أخرى مشبوهة ذات تعزيز في الربع العلوي الداخلي في الثلث الأمامي من الغدة، بقياس 0.5 سم (سلسلة 16، صورة رقم 83).
لا يوجد تعزيز مشبوه يتوافق مع نتائج السونار السابق في الجزء الداخلي (جهة منتصف الصدر).
يوجد مشبك معدني (بعد الخزعة) في الربع المركزي الداخلي دون وجود تعزيز مشبوه تحته، وهو يتوافق مع أنسجة الثدي الحميدة المثبتة بالخزعة في الجزء السفلي الخارجي (جهة الإبط).
بؤر تعزيز متدرجة في المنطقة الواقعة خلف الحلمة مباشرة، بقياس 0.4 سم (سلسلة 13، صورة رقم 58).
لا يوجد سماكة في الجلد أو تراجع في الحلمة.
تعدد عقد لمفاوية مشبوهة في الإبط الأيسر تشمل بشكل أساسي المستوى الأول (Level 1)، أكبرها بقياس 1.9 × 1 سم.
لا توجد عقد لمفاوية متضخمة داخل الثدي.

IMPRESSION: (الخلاصة)

- ورم خبيث مثبت بالخزعة في الثدي الأيسر في الجزء السفلي من الثدي، مع وجود عقدتين مشبوهتين إضافيتين في الجزء السفلي خلف الكتلة وفي الربع العلوي الداخلي؛ يُنصح بإعادة فحص السونار وأخذ خزعة تحت إرشاد السونار لاستبعاد وجود المرض في مراكز متعددة من الثدي.
- عقد لمفاوية إبطية في نفس الجانب منتشر إليها الورم بوضوح.

.BI-RADS: 6, Known Biopsy-Proven Malignancy

(خباثة مؤكدة بالخزعة)

هل ترغب في أن أشرح لك أيًا من المصطلحات الطبية المذكورة في التقرير أو أوضح لك الخطوات القادمة المقترحة بشكل أبسط؟

Report Number # 1

MRN 8100031284. DATE June 27, 2021
MODALITY: MAMMOGRAM—DIAGNOSTIC & ULTRASOUND BREAST—BILATERAL:
CLINICAL INDICATION:
Right breast nodule seen on recent CT scan.

FINDINGS ON MAMMOGRAM:
Heterogeneous fibroglandular densities may obscure small pathology.
There is focal asymmetry in the right breast around the 6 o'clock axis. It is partially included due to the deep location and overlaps a circumscribed oval nodule seen in exaggerated medial CC view. No associated microcalcifications in the provided images. It partially persisted on spot compression view; however, evaluation remains suboptimal due to the posterior location of the finding. No nipple retraction or suspicious skin thickening.
On the left side, there are multiple mildly enlarged axillary lymph nodes with mild stranding of the surrounding fat plane. The patient gave a history of recent COVID-19 vaccination taken in the left arm.

FINDINGS ON ULTRASOUND:
The circumscribed oval hypoechoic nodule in right breast at 12 o'clock measures 1.0 x 0.5 x 1.4 cm; it likely corresponds to the mass described on CT scan.
A smaller likely similar nodule noted in the right breast at 8 o'clock.
No suspicious masses or abnormality correlates with the focal asymmetry at 6 o'clock.
Abnormal appearance of left axillary lymph nodes with mild uniform borderline cortical thickening likely reactive.
IMPRESSION:
Non-palpable benign-looking right breast mass at 12 o'clock likely corresponds to the finding on CT scan and follow-up recommended.
Partially included deeply located focal asymmetry in the right breast close to the 6 o'clock axis without corresponding suspicious masses on ultrasound; the findings may represent focal asymmetric glandular. Differential diagnosis includes radial scar or less likely malignancy; further evaluation by MRI is recommended.

BI-RADS 0.

“" "Report Number # 1

MRN 8100031284- DATE June 27, 2021
MODALITY: MAMMOGRAM – DIAGNOSTIC & ULTRASOUND BREAST -BILATERAL:
CLINICAL INDICATION:
عُقيدة في الثدي الأيمن شوهدت في تصوير CT حديث.
FINDINGS ON MAMMOGRAM:
كثافات ليفية غدية غير متجانسة قد تُخفي آفات صغيرة.
يوجد عدم تناظر بؤري في الثدي الأيمن في الجزء السفلي قرب منطقة الحلمة. تم تضمينه جزئيًا فقط بسبب عمقه وتراكبه مع عُقيدة بيضاوية محددة الحواف شوهدت في صورة CC مع تكبير باتجاه الجانب الداخلي. لا توجد تكلسات دقيقة مرافقة في الصور المتاحة. استمر جزئيًا في صورة الضغط الموضعي(spot compression) ، إلا أن التقييم ما يزال غير مثالي بسبب الموقع الخلفي للعثور. لا يوجد ارتداد بالحلمة ولا سماكة جلدية مشتبهة.
في الجانب الأيسر، توجد عدة عقد لمفاوية إبطيّة متضخمة بشكل خفيف مع خطوط/تغيرات خفيفة في الدهون المحيطة.
وذكرت المريضة تاريخ أخذ لقاح COVID حديثًا في الذراع الأيسر.
FINDINGS ON ULTRASOUND:
عُقيدة بيضاوية محددة الحواف ناقصة الصدى (hypoechoic) في الثدي الأيمن في الجزء العلوي قرب منطقة الحلمة، قياسها 1.0 x 0.5 x 1.4 cm ، ومن المرجح أنها تقابل الكتلة الموصوفة في تصوير.CT
لوحظت عُقيدة أصغر يُحتمل أنها مشابهة في الثدي الأيمن في الجزء الخارجي (باتجاه الإبط) من الثدي.
لا توجد كتل مشتبهة أو شذوذ يُطابق عدم التناظر البؤري في الثدي الأيمن في الجزء السفلي قرب منطقة الحلمة.
مظهر غير طبيعي للعقد اللمفاوية الإبطية اليسرى مع زيادة خفيفة منتظمة وحدّية في سماكة القشرة، ويرجّح أن يكون تفاعليًا.
IMPRESSION:
كتلة في الثدي الأيمن غير محسوسة باليد وتبدو حميدة في الجزء العلوي قرب منطقة الحلمة، ويرجّح أنها تقابل ما شوهد في تصويرCT ، ويوصى بالمتابعة.
عدم تناظر بؤري عميق ومُضمَّن جزئيًا في الثدي الأيمن في الجزء السفلي قرب منطقة الحلمة دون وجود كتل مشتبهة مقابلة فيUltrasound ؛ قد تمثل هذه النتائج نسيجًا غديًا غير متناظر بؤريًا. التشخيصات التفريقية تشمل radial scar أو وبدرجة أقل خباثة؛ يُوصى بمزيد من التقييم بواسطة.MRI
BI-RADS 0.) غير مكتمل — يحتاج تصويرًا إضافيًا للتقييم و/أو صورًا سابقة للمقارنة حسب الفحص(
"
”

Report Number # 2

Follow-up of former patient in March 21, 2023
Mammogram diagnostic

CLINICAL DATA:
LCIS post excision for mammogram

FINDINGS:
Study compared to prior mammogram performed in June 2021.
Heterogenous fibroglandular densities.
Status post excision of focal asymmetry in right breast associated with distortion.
Mild postoperative distortion noted on Tomosynthesis.
No developing masses or suspicious microcalcifications.
No nipple retraction or suspicious skin thickening.
Stable appearance of the axillary lymph nodes.

IMPRESSION:
Status post excision of the right-side radial sclerosing pathology with lobular neoplasia.
For risk appropriate screening.
BI-RADS 2

"Report Number # 2
Follow up of former patient in March 21, 2023
Mammogram diagnostic
CLINICAL DATA:
LCIS post excision for mammogram
FINDINGS:
Study compared to prior mammogram done in June 2021
كثافات ليفية غدية غير متجانسة
حالة ما بعد الاستئصال لعدم تناظر بؤري في الثدي الأيمن مرتبط بتشوّه/اختلال في البنية.
يوجد تشوّه خفيف بعد العملية يظهر في.Tomosynthesis
لا توجد كتل جديدة متشكّلة أو تكلسات دقيقة مشتبه بها.
لا يوجد تراجع في الحلمة ولا يوجد تثخّن جلدي مشتبه به.
مظهر العقد اللمفاوية الإبطية ثابت دون تغيّر.
IMPRESSION:
حالة ما بعد استئصال آفة تصلّب شعاعي في الجهة اليمنى مع ورم/تغيّرات فصيصية.(lobular neoplasia)
لإجراء فحص تحرّي/مسح مناسب بحسب درجة الخطورة.
BI-RADS 2: (حميد)
"

Report Number # 3

MRN 72591589. DATE: Nov, 11 2025

Ultrasound breast

INDICATIONS:
Breast cancer on hormonal therapy; last mammogram showed BI-RADS 3.

FINDINGS:
There is a circumscribed oval mass in left breast at 6:00, 2 cm from nipple, with nonseparable tiny smaller similar masses/cysts measuring collectively 1.3 x 0.3 cm. No significant interval change as compared to ultrasound performed in May 2025 and November 2023.
Circumscribed oval heterogenous isoechoic area/mass at 6:00, 5 cm from the nipple, measures 1.8 x 0.6 cm without significant change. It likely represents focal prominent fat tissue rather than a genuine lesion. Smaller similar area noticed laterally.
No associated signs of invasion or enlarged lymph nodes.
Right-side mastectomy with signs of axillary dissection.

IMPRESSION:
Stable small mass at 6:00 for more than 2 years.
The additional findings in the same region indicate likely prominent fat lobules rather a genuine lesion.
Follow-up by ultrasound after 6 months is recommended [patient will be due for annual mammographic assessment in May 2026].

BI-RADS 3

Report Number # 3
MRN 72591589 DATE: Nov, 11 2025
Ultrasound breast
INDICATIONS:
سرطان الثدي على علاج هرموني، وآخر تصوير ماموغرام أظهر BI-RADS 3.
FINDINGS:
توجد كتلة بيضوية محددة الحواف في الثدي الأيسر في **الجزء السفلي من الثدي قرب منطقة الحلمة**، على بُعد 2 cm من الحلمة، مع كتل/أكياس صغيرة جدًا مشابهة وغير منفصلة عنها، ويبلغ قياسها مجتمعة 1.3 x 0.3 cm. لا يوجد تغير زمني مهم مقارنةً بفحص الموجات فوق الصوتية في May 2025 وNovember 2023.
توجد منطقة/كتلة بيضوية محددة الحواف غير متجانسة ومتساوية الصدى (isoechoic) في الثدي الأيسر في **الجزء السفلي من الثدي قرب منطقة الحلمة**، على بُعد 5 cm من الحلمة، ويبلغ قياسها 1.8 x 0.6 cm دون تغير مهم. يُرجَّح أنها تمثل نسيجًا دهنيًا بارزًا موضعيًا أكثر من كونها آفة حقيقية. لوحظت منطقة أصغر مشابهة على **الجانب الخارجي من الثدي (باتجاه الإبط.(**
لا توجد علامات مرافقة للغزو ولا توجد عقد لمفاوية متضخمة.
استئصال الثدي في الجهة اليمنى مع علامات تشريح/تفريغ إبطي (axillary dissection).
IMPRESSION:
كتلة صغيرة مستقرة في **الجزء السفلي من الثدي** لأكثر من فترة 2 years.
النتائج الإضافية في نفس المنطقة يُرجَّح أنها فصيصات دهنية بارزة أكثر من كونها آفة حقيقية.
يوصى بالمتابعة بالموجات فوق الصوتية بعد 6 months [سيحين موعد التقييم السنوي بالماموغرام في May 2026].
BI-RADS 3: (غالبًا حميد — يُنصح عادةً بالمتابعة)

Report Number # 4

MRN: 13024229. DATE: Feb 20, 2025
MRI breast
INDICATIONS:
Suspicious breast lesion requires better assessment and evaluation
TECHNIQUE:
Multiple sequential multiplanar MRI breast with dynamic post-contrast study.
FINDINGS: There is an irregular mass with irregular margins within the nipple and subareolar region measuring 1.3 x 1.3 x 1.7 cm. It contains a central round signal void area representing the postbiopsy marker. It is heterogenous in signal intensity, predominantly isointense, and demonstrates marked rapid initial enhancement with washout.

Another mass with similar features noticed in the upper outer quadrant of the left breast measures 1.2 x 0.8 x 1 cm. It is about 4 cm away from the nipple. An additional small irregular mass with irregular margins noticed at a lower aspect close to the 6:00 position/central retroareolar region about 5 cm from nipple [image 85 in post-contrast series].

Segmental heterogenous nonmass enhancement noticed in the left upper outer quadrant and extending from the retroareolar region and 12:00 position reaching the retromammary prepectoral fascia containing subcentimeter rapidly enhancing masses and duct ectasia, worrisome for similar pathology and/or associated in situ carcinoma.

On the right side, at the outer aspect close to the 9:00 position, there is a deeply located subcentimeter irregular rapidly enhancing mass measuring 0.5 x 0.5 cm and demonstrating washout enhancement. Multiple small cysts noticed in the same area.

Background heterogenous mammary tissue with the heterogenous mild-to-moderate background parenchymal enhancement.

Bilateral subcentimeter cysts noticed and duct ectasia.

The nipple areolar complex on the right side is unremarkable.

No suspicious axillary lymph nodes.

IMPRESSION:

Mass within the left nipple and subareolar region highly suggestive of malignancy containing postbiopsy marker.

Additional masses and nonmass enhancements as described in the ipsilateral breast likely similar in nature and/or associated in situ disease.

On the right side, there is a small suspicious mass.

BI-RADS 4

Report Number # 4

MRN: 13024229 DATE: Feb 20, 2025

MRI breast

INDICATIONS:

آفة مشتبه بها في الثدي لمزيد من التقييم والدراسة بشكل أفضل.

TECHNIQUE:

تصوير MRI للثدي متعدد المقاطع وبمستويات متعددة، مع دراسة ديناميكية بعد حقن المادة المتباينة.

FINDINGS:

توجد كتلة غير منتظمة ذات حواف غير منتظمة داخل منطقة الحلمة وما تحت الحلمة، قياسها 1.3 x 1.3 x 1.7 cm. تحتوي على منطقة مركزية دائرية خالية من الإشارة تمثل علامة ما بعد الخزعة (postbiopsy marker). الإشارة غير متجانسة وتميل لأن تكون متساوية الشدة (isointense) بشكل غالب، وتُظهر تعزيزًا أوليًا سريعًا وملحوظًا مع نمط غسيل (washout).

كما توجد كتلة أخرى بصفات مشابهة في الربع العلوي الخارجي من الثدي الأيسر قياسها 1.2 x 0.8 x 1 cm، وتبعد حوالي 4 cm عن الحلمة. توجد أيضًا كتلة صغيرة إضافية غير منتظمة ذات حواف غير منتظمة في الجزء السفلي من الثدي الأيسر، قريبة من المنطقة المركزية خلف الحلمة/الهالة(retroareolar) ، وتبعد حوالي 5 cm عن الحلمة [image 85 in post contrast series].

يوجد تعزيز غير كتلي (nonmass enhancement) قطعي (segmental) غير متجانس في الربع العلوي الخارجي من الثدي الأيسر، ويمتد من المنطقة خلف الهالة/الحلمة ومن الجزء العلوي من الثدي وصولًا إلى اللفافة خلف الثدي أمام العضلة الصدرية(retromammary prepectoral fascia) ، ويحتوي على كتل صغيرة سريعة التعزيز دون

السنتيمتر مع توسع قنوات(duct ectasia) ، وهو مقلق لاحتمال نفس المرض و/أو سرطان موضعي مرافق (in situ carcinoma).
في الثدي الأيمن، في الجانب الخارجي (باتجاه الإبط) توجد كتلة غير منتظمة صغيرة دون السنتيمتر عميقة الموضع وسريعة التعزيز قياسها 0.5 x 0.5 cm وتُظهر نمط غسيل .(washout) لوحظت عدة أكياس صغيرة في نفس المنطقة.
يوجد نسيج ثديي خلفي غير متجانس مع تعزيز خلفي (background parenchymal enhancement) غير متجانس خفيف إلى متوسط.
لوحظت أكياس ثنائية الجانبين دون السنتيمتر وتوسع قنوات.(duct ectasia)
مركّب الحلمة والهالة في الثدي الأيمن دون ملاحظات غير طبيعية.
لا توجد عقد لمفاوية إبطيّة مشتبه بها.
IMPRESSION:
كتلة ضمن منطقة الحلمة وما تحت الحلمة في الثدي الأيسر مرجّحة بشدة للخباثة وتحتوي على علامة ما بعد الخزعة (postbiopsy marker).
كتل إضافية وتعزيزات غير كتلية كما وُصفت في نفس الثدي يُرجّح أنها مشابهة بالطبيعة و/أو مرض موضعي مرافق (in situ).
كتلة صغيرة مشتبه بها في الثدي الأيمن.
BI-RADS 4: (مشتبه)

Report Number # 5

MRN: Missed. DATE: April 7, 2025
MRI OF THE BREAST
CLINICAL INDICATION:
A 45-year-old female patient diagnosed with left breast cancer requires better assessment and evaluation.
Additional history:
Patient has multiple ultrasound-guided biopsies as follows:
1. Right breast, mass at 3 o'clock, core needle biopsy:
- Fibroadenoma.
2. Left breast, mass at 6 o’clock, core needle biopsy:
- Invasive ductal carcinoma of no special type, mSBR grade 3 (tubular formation 3, nuclear pleomorphism 3, mitotic rate 2).
3. Left breast, mass at 8 o’clock, core needle biopsy:
- Benign breast tissue.

TECHNIQUE:
Multiplanar multisequential MRI of the breasts was performed prior to and following the administration of IV contrast gadolinium utilizing routine tumor protocol.
COMPARISON:
None. Correlation was made with mammogram and ultrasound dated February 26, 2025.
FINDINGS:
Almost entirely fat (ACR type A).
Mild symmetric background parenchymal enhancement.

RIGHT BREAST:
There is a circumscribed oval-shaped minimally enhancing lesion in the right breast central inner quadrant of the anterior third of the gland measuring 1.3 x 0.8 cm with an internal postbiopsy clip which corresponds to biopsy-proven fibroadenoma.
No suspicious mass or nonmass enhancement.
No skin thickening or nipple retraction.
LEFT BREAST:
There is a spiculated heterogeneously enhancing mass in the left hip breast lower central quadrant of the mid-third of the gland, measuring 2 x 2.3 x 1.9 cm (TV, AP and craniocaudal) with an internal postbiopsy clip, which corresponds to biopsy-proven malignancy.
Just 4.8 cm posterior and inferior to the index mass, there is another irregular enhancing nodule close to the cell, measuring 0.5 cm, worrisome for multifocal disease (series 16, image #76).
Another suspicious enhancing nodule in the upper inner quadrant of the anterior third of the gland measures 0.5 cm (series 16, image #83).
No suspicious enhancement corresponding to findings on prior ultrasound at 3:00.
Postbiopsy clip in the central inner quadrant without underlying suspicious enhancement which corresponds to biopsy-proven benign breast tissue at 8:00.
Progressive foci of enhancement in the subareolar region measure 0.4 cm (series 13, image #58).
No skin thickening or nipple retraction.
Multiple suspicious left axillary lymph nodes mainly involving level 1. The largest measures 1.9 x 1 cm.
No enlarged intramammary lymph nodes.
IMPRESSION:
- Biopsy-proven malignancy in the left breast at 6 o'clock with 2 additional suspicious nodules at 6 o'clock posteriorly and in the upper inner quadrant; for a second look, ultrasound and ultrasound-guided biopsy are required to exclude multicentric disease.
- Clearly metastatic ipsilateral axillary lymph nodes.
BI-RADS: 6, Known Biopsy-Proven Malignancy.

Report Number # 5
MRN: مفقودDATE: April 7, 2025 .
MRI OF THE BREAST
CLINICAL INDICATION:
مريضة عمرها 45 سنة تم تشخيصها بسرطان الثدي الأيسر، وذلك لتقييم الحالة بصورة أفضل.
Additional history:
لدى المريضة عدة خزعات موجَّهة بالموجات فوق الصوتية كما يلي:

1. Right breast, كتلة في الربع الداخلي الأمامي باتجاه مركز الثدي (نحو منتصف الصدر) ضمن الثدي الأيمن، خزعة بإبرة أساسية:(core needle biopsy)
   - ورم ليفي غدي.(Fibroadenoma)
2. Left breast, كتلة في الجزء السفلي من الثدي الأيسر (بالقرب من منطقة الحلمة/أسفلها)، خزعة بإبرة أساسية (core needle biopsy):
   - سرطانة قَنَوية غازية من نوع غير خاص(Invasive ductal carcinoma of no special type) ، درجة mSBR 3 (تشكّل أنبوبي 3، تَعدُّد أشكال النوى 3، معدل الانقسام 2.)

3. كتلة في الجزء السفلي الخارجي من الثدي الأيسر (باتجاه الإبط)، خزعة بإبرة أساسية (core Left breast, needle biopsy):
   - نسيج ثدي حميد.

TECHNIQUE:

تم إجراء MRI للثديين بمستويات متعددة وتسلسلات متعددة قبل وبعد إعطاء مادة تباين غادولينيوم عبر الوريد باستخدام بروتوكول الأورام الروتيني.

COMPARISON:

لا يوجد. تمت المقارنة مع تصوير الثدي بالأشعة (mammogram) والموجات فوق الصوتية بتاريخ February 26, 2025.

FINDINGS:

الثديان شبه دهنيين بالكامل.(ACR type A)

تعزّز خفيف متناظر في نسيج الثدي الخلفي.(background parenchymal enhancement)

RIGHT BREAST:

توجد آفة بيضاوية محددة الحواف مع تعزز بسيط جدًا في الربع الداخلي المركزي من الثدي الأيمن ضمن الثلث الأمامي من الغدة، قياسها 1.3 x 0.8 cm ، وبداخلها مشبك (clip) بعد الخزعة، وهذا يتوافق مع ورم ليفي غدي مُثبت بالخزعة.

لا توجد كتلة مشتبه بها أو تعزز غير كتلي مشتبه به.

لا يوجد تثخّن بالجلد أو تراجع بالحلمة.

LEFT BREAST:

توجد كتلة شوكية (spiculated) مع تعزز غير متجانس في الربع السفلي المركزي من الثدي الأيسر ضمن الثلث الأوسط من الغدة، قياسها 2 x 2.3 x 1.9 cm (TV, AP and craniocaudal) ، وبداخلها مشبك (clip) بعد الخزعة، ويتوافق ذلك مع خباثة مثبتة بالخزعة.

على بُعد 4.8 cm خلفيًا وأسفلًا من الكتلة الأساسية(index mass) ، توجد عقدة أخرى غير منتظمة متعززة قريبة من الـcell، قياسها 0.5cm ، مما يثير القلق لوجود مرض متعدد البؤر.(series 16, image #76)

كما توجد عقدة أخرى مشتبه بها متعززة في الربع العلوي الداخلي ضمن الثلث الأمامي من الغدة، قياسها 0.5 cm (series 16, image #83).

لا يوجد تعزز مشتبه به يتوافق مع الموجودات في الموجات فوق الصوتية السابقة في الجانب الداخلي من الثدي الأيسر (باتجاه منتصف الصدر.(

يوجد مشبك (clip) بعد الخزعة في الربع الداخلي المركزي دون تعزز مشتبه به تحته، ويتوافق ذلك مع نسيج ثدي حميد مُثبت بالخزعة في الجزء السفلي الخارجي من الثدي الأيسر (باتجاه الإبط.(

بؤر متزايدة التعزز في المنطقة تحت الهالة(subareolar region) ، قياسها 0.4.cm (series 13, image #58)

لا يوجد تثخّن بالجلد أو تراجع بالحلمة.

عدة عُقَد لمفاوية إبطيّة يسرى مشتبه بها، ومعظمها في المستوى 1. أكبرها قياسه 1.9 x 1 cm.

لا توجد عُقَد لمفاوية داخل الثدي (intramammary) متضخمة.

IMPRESSION:

-خباثة مثبتة بالخزعة في الثدي الأيسر في الجزء السفلي من الثدي (بالقرب من منطقة الحلمة/أسفلها) مع وجود عقدتين إضافيتين مشتبه بهما في الجزء السفلي من الثدي الأيسر خلفيًا، وفي الربع العلوي الداخلي؛ ويوصى بإجراء موجات فوق صوتية (second look ultrasound) وخزعة موجَّهة بالموجات فوق الصوتية لاستبعاد وجود مرض متعدد المراكز (multicentric disease).

-عُقَد لمفاوية إبطيّة على نفس الجهة (ipsilateral) مع انتقالات واضحة.

BI-RADS: 6, Known Biopsy-Proven Malignancy. (خباثة مؤكدة بالخزعة)
