# Supplementary material for: Large Language Model Translation of BI-RADS Breast Imaging Reports into Arabic: A Blinded Expert Evaluation of Diagnostic Communication Safety
Source: Diagnostics (Basel). 2026 Aug 7;16(16):2495. doi: 10.3390/diagnostics16162495 (PMC13510894; doi:10.3390/diagnostics16162495)
Supplement: Supplementary file 1 [file diagnostics-16-02495-s001.zip › Supplementary Material S2.pdf]

Supplementary Material 2: Characteristics of five source breast imaging reports used for LLM translation evaluation

Report	BI-RADS	Modality	Laterality	Lesion type/finding	Measurements reported	Word count	Recommendation
1	0	Diagnostic mammogram + bilateral ultrasound	Bilateral (primary finding right; reactive nodes left)	Circumscribed oval nodule, likely benign, corresponding to CT finding; separate deep focal asymmetry, indeterminate (differential: radial scar vs. malignancy); reactive left axillary lymphadenopathy	1 (1.0 × 0.5 × 1.4 cm)	272	Further evaluation by MRI recommended
2	2	Diagnostic mammogram (follow-up)	Right (post-excision)	Postoperative distortion status post excision of radial sclerosing lesion with lobular neoplasia (LCIS); no active mass	0	94	Risk-appropriate screening
3	3	Breast ultrasound	Bilateral (active findings left; right status post mastectomy)	Circumscribed oval mass with small associated cysts, stable ≥2 years; separate area favored as fat lobulation rather than true lesion	2 (1.3 × 0.3 cm; 1.8 × 0.6 cm)	170	Short-interval (6-month) ultrasound follow-up
4	4	Breast MRI	Bilateral (multifocal left; small right lesion)	Multiple irregular enhancing masses and segmental nonmass enhancement, left breast, highly suggestive of malignancy, with postbiopsy marker; additional small suspicious right-sided mass	3 (1.3 × 1.3 × 1.7 cm; 1.2 × 0.8 × 1.0 cm; 0.5 × 0.5 cm)	294	Tissue diagnosis (biopsy) per BI-RADS 4 category
5	6	Breast MRI	Bilateral (known malignancy left; benign right)	Biopsy-proven invasive ductal carcinoma (left) with 2 additional suspicious nodules and suspicious axillary lymphadenopathy; biopsy-proven fibroadenoma and benign tissue (right)	6 (1.3 × 0.8 cm; 2 × 2.3 × 1.9 cm; 0.5 cm; 0.5 cm; 0.4 cm; 1.9 × 1 cm)	407	Second-look ultrasound and ultrasound-guided biopsy to exclude multicentric disease
